# 10*H*-1,9-diazaphenothiazine and its 10-derivatives: synthesis, characterisation and biological evaluation as potential anticancer agents

**DOI:** 10.1080/14756366.2019.1639695

**Published:** 2019-07-16

**Authors:** Beata Morak-Młodawska, Krystian Pluta, Małgorzata Latocha, Małgorzata Jeleń, Dariusz Kuśmierz, Kinga Suwińska, Aleksander Shkurenko, Zenon Czuba, Magdalena Jurzak

**Affiliations:** a Department of Organic Chemistry, School of Pharmacy with the Division of Laboratory Medicine, The Medical University of Silesia, Sosnowiec, Poland;; b Department of Cell Biology, School of Pharmacy with the Division of Laboratory Medicine, The Medical University of Silesia, Sosnowiec, Poland;; c Faculty of Mathematics and Natural Sciences, Cardinal Stefan Wyszyński University, Warszawa, Poland;; d A. M. Butlerov Institute of Chemistry, Kazan Federal University, Kazan, Russia;; e Division of Physical Functional Materials Design, Discovery & Development Research Group (FMD3), Sciences and Engineering Advanced Membranes & Porous Materials (AMPM), King Abdullah University of Science and Technology (KAU ST), Thuwal, Kingdom of Saudi Arabia;; f Department of Microbiology and Immunology, Medical University of Silesia in Katowice, Zabrze, Poland

**Keywords:** Phenothiazines, dipyridothiazines, anticancer activity, gene expressions, 2D NMR spectra

## Abstract

10*H*-1,9-diazaphenothiazine was obtained in the sulphurisation reaction of diphenylamine with elemental sulphur and transformed into new 10-substituted derivatives, containing alkyl and dialkylaminoalkyl groups at the thiazine nitrogen atom. The 1,9-diazaphenothiazine ring system was identified with advanced ^1^H and ^13^C NMR techniques (COSY, NOESY, HSQC and HMBC) and confirmed by X-ray diffraction analysis of the methyl derivative. The compounds exhibited significant anticancer activities against the human glioblastoma SNB-19, melanoma C-32 and breast cancer MDA-MB-231 cell lines. The most active 1,9-diazaphenothiazines were the derivatives with the propynyl and *N*, *N*-diethylaminoethyl groups being more potent than cisplatin. For those two compounds, the expression of *H3*, *TP53*, *CDKN1A*, *BCL-2* and *BAX* genes was detected by the RT-QPCR method. The proteome profiling study showed the most probable compound action on SNB-19 cells through the intrinsic mitochondrial pathway of apoptosis. The 1,9-diazaphenotiazine system seems to be more potent than known isomeric ones (1,6-diaza-, 1,8-diaza-, 2,7-diaza- and 3,6-diazaphenothiazine).

## Graphical Abstract

**Figure:**
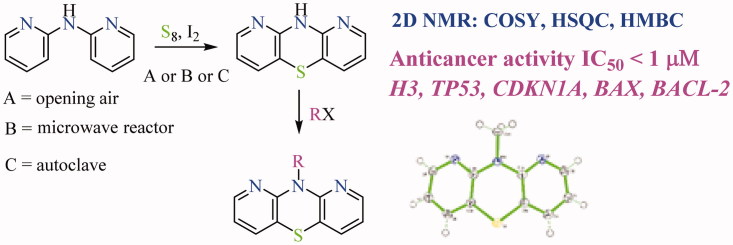

## Introduction

In the last decades, cancer has been one of the main causes of death worldwide affecting millions of people per year. The main forms of a curative treatment for tumours are surgery, radiation, chemotherapy and biotherapy^1^^,^^2^. The goal of chemotherapeutic agents is to cure the tumour, to prolong survival and to reduce the tumour burden to alleviate symptoms. In recent years, a lot of effort has been applied to the synthesis of potential anticancer drugs with better selectivity and minor or no side effects^1–4^.

Synthetical and natural bioactive compounds with heterocyclic ring systems play an important role for the development of novel scaffolds in medicinal chemistry^5^^,^^6^. One of the most active heterocyclic rings is a 1,4-thiazine ring, containing the nitrogen and sulphur atoms^7^. This ring fused with two benzene rings forms a dibenzothiazine system, present in one of most valuable drugs – phenothiazines. Classical phenothiazines with the dialkylaminoalkyl groups at the nitrogen atom (and additional simple group at the carbon atom in position 2) have still been recognised as neuroleptic, antihistaminic, antitussive and antiemetic drugs^8^. Recently, many papers were published revealing new activities for these compounds, for example, thioridazine, one of the most known phenothiazines, exhibits promising properties for multidrug-resistant tuberculosis treatment^9^ and lung cancer therapy through targeting lung cancer stem cells, due to its efficacy and safety^10^.

On the other hand, the phenothiazine structure has been modified mainly by introduction of new substituents at the thiazine nitrogen atom and by replacement of one or two benzene rings with various azine rings (leading to azaphenothiazines). Recent numerous original reports, reviews and chapters in monographs describe new promising biological activity of both classical and modified phenothiazines such anticancer, anti-plasmid, antiviral, anti-inflammatory and antibacterial activities, reversal of multi-drug resistance^8^^,^^11–20^. They are promising candidates for further studies directed to the development of new drugs useful in the treatment of Creutzfeldt-Jakob’s, Alzheimer’s and other neurodegenerative diseases, like amyotrophic lateral sclerosis, Parkinson’s and Huntington’s diseases^8^^,^^21–23^.

The modifications of the phenothiazine structure with one or two pyridine rings lead to pyridobenzothiazines and dipyridothiazines, respectively. Of the four isomeric pyridobenzothiazines (being 1-aza-, 2-aza-, 3-aza- and 4-azaphenothiazines), the 10-dialkylaminoalkyl derivatives of the 1-aza series (known as prothipendyl, isothipendyl, oxypendyl, cloxypendyl and pipazethate) are still used in the various therapies such as antipsychotic^24–26^, antihistaminic^27^^,^^28^, and antitussive^29^ and antiemetic drugs^30^.

Dipyridothiazines are less known and out of 10 possible isomeric types only 6 types have been synthesised: 1,6-diaza-, 1,8-diaza-, 1,9-diaza-, 2,7-diaza-, 3,6-diaza- and 3,7-diazaphenothiazines. Initially, only 1,6-diaza- and 3,7-diazaphenothiazines were examined for their biological activities: neuroleptic^31^ and antihistaminic^32^. Recently, some derivatives of four types of dipyridothiazines (1,6-diaza-, 1,8-diaza-, 2,7-diaza- and 3,6-diazaphenothiazines) were found to exhibit potent anticancer activities, stronger than cisplatin, against various types of cancer cell lines^33–39^. 1,8-Diaza- and 2,7-diazaphenothiazines showed strong inhibition of tumour necrosis factor alpha production induced by lipopolysaccharide^33^^,^^37^^,^^38^. 10H-2,7-diazaphenothiazine was very strongly suppressive with regard to the secondary humoral response *in vitro* and significantly inhibited the delayed-type hypersensitivity response to ovalbumin *in vivo* in mice^37^. Its isomer, 10H-3,6-diazaphenothiazine very recently was found to possess potential as a chemotherapeutic agent with cytostatic and cell cycle inhibiting actions^39^.

The aim of this paper is elaboration of efficient synthesis of 10*H*-1,9-diazaphenothiazine, transformation this compound into 11 varied 10-substituted derivatives and determination of their anticancer activity against selected tumour cell lines. Since the synthesis of the parent compound was only mentioned in a patent^40^ without any details, the elaboration of efficient method of preparation is crucial challenge.

## Materials and methods

### Chemistry

Melting points were determined in open capillary tubes on a Boetius melting point apparatus and are uncorrected. The ^1^H NMR, COSY, ROESY, HSQC, HMBC spectra were recorded on an Ascend^TM^ 600 spectrometers at 600 MHz in deuteriochloroform with tetramethylsilane as the internal standard. The ^13^C NMR spectrum was recorded at 75 MHz. Electron impact mass spectra (EI MS), fast atom bombardment mass spectra (FAB MS, in glycerol), chemical ionisation (CI MS) were run on a Finnigan MAT 95 spectrometer at 70 eV and HR MS was run on a Brucker Impact II. The thin layer chromatography was performed on silica gel 60 F_254_ (Merck 1.05735) with CHCl_3_-EtOH (10:1 v/v) and on aluminium oxide 60 F_254_ neutral (type E) (Merck 1.05581) with CHCl_3_-EtOH (10:1 v/v) as eluents.

#### Synthesis of 10H-1,9-diazaphenothiazine (2) through sulphurisation of 2,2′-dipyridylamine (1) with elemental sulphur

In open air conditions. A mixture of 2,2′-dipyridinylamine (**1**) (0.171 g, 1 mmol), elemental sulphur (0.064 g, 2 mmol) and small crystal of iodine was heated at 250 °C for 1 h. After cooling the reaction mixture was extracted with CHCl_3_ (3 × 10 ml). The obtained product was purified by column chromatography (aluminium oxide, CHCl_3_) to give 10*H*-1,9-diazaphenothiazine (**2**) (0.056 g, 28%); mp 156–157 °C.

^1^H NMR (CDCl_3_) δ: 6.74 (dd, *J* = 7.8 Hz, *J* = 4.8 Hz, 2H, H_3,_ H_7_), 7.16 (dd, *J* = 7.8 Hz, *J* = 1.8 Hz, 2H, H_4,_ H_6_), 7.96 (dd, *J* = 4.8 Hz, *J* = 1.8 Hz, 2H, H_2_, H_8_), 8.33 (s, 1H, NH). ^13^C NMR (CDCl_3_): 112.86 (C_4a_, C_5a_), 118.61 (C_3_, C_7_), 133.53 (C_4_, C_6_), 145.91 (C_2_, C_8_), 152.35 (C_9a_, C_10a_). EI MS *m*/*z*: 201 (M, 100). HR MS (EI) *m*/*z* calc. for [C_10_H_7_N_3_S + H] 202.0439. Found 202.0455.

In a microwave reactor. A mixture of 2,2′-dipyridinylamine (**1**) (0.171 g, 1 mmol), elemental sulphur (0.064 g, 2 mmol) and small crystal of iodine was added to the clean quartz reactor vessel. The mixture was heated at 230 °C during 30 min. The obtained brown oil was purified by column chromatography (aluminium oxide, CHCl_3_) to give 10*H*-1,9-diazaphenothiazine (**2**) (0.083 g, 42%).

In an autoclave. A mixture of 2,2′-dipyridinylamine (**1**) (0.171 g, 1 mmol), elemental sulphur (0.064 g, 2 mmol) and small crystal of iodine in dioxane (5 ml) was added to the clean autoclave. The mixture was heated at 230 °C, under pressure of 17 bar, during 2 h. The obtained brown oil was evaporated *in vaccuo*. The dry residue was dissolved in CHCl_3_ and purified by column chromatography (aluminium oxide, CHCl_3_) to give 10H-1,9-diazaphenothiazine (**2**) (0.034 g, 17%).

#### Synthesis of 10-substituted 1,9-diazaphenothiazines (3–5)

To a solution of 10*H*-1,9-diazaphenothiazine (**2**) (0.50 g, 0.25 mmol) in dry DMF (5 ml) NaH (0.012 g, 0.5 mmol, 60% NaH in mineral oil was washed out with hexane) was added. The reaction mixture was stirred at room temperature for 1 h and then alkyl or aryl halides (methyl iodide, allyl bromide, benzyl chloride, 0.75 mmol) were added and the stirring was continued for 24 h. The mixture was poured into water (15 ml), extracted with CHCl_3_ (3 × 10 ml) and dried using Na_2_SO_4_. The obtained product was purified by column chromatography (aluminium oxide, CHCl_3_) to give the following:

10-Methyl-1,9-diazaphenothiazine (**3**) (0.041 g, 75%); mp 122–123 °C.

^1^H NMR (CDCl_3_) δ: 3.45 (s, 3H, CH_3_), 6. 81 (dd, *J* = 7.8 Hz, *J* = 4.8 Hz, 2H, H_3_, H_7_), 7.26 (dd, *J* = 7.8 Hz, *J* = 1.8 Hz, 2H, H_4_, H_6_), 8.08 (dd, *J* = 4.8 Hz, *J* = 1.8 Hz, 2H, H_2_, H_8_). ^13^C NMR (CDCl_3_): 31.50 (CH_3_), 115.48 (C_4a_, C_5a_), 118.16 (C_3_, C_7_), 133.83 (C_4,_ C_6_), 145.55 (C_2_, C_8_), 154.26 (C_9a_, C_10a_). EI MS *m*/*z*: 215 (M, 100). HR MS (EI) *m*/*z* calc. for [C_11_H_9_N_3_S + H] 216.0595. Found 216.0597.

10-Allyl-1,9-diazaphenothiazine (**4**) (0.045 g, 75%); an yellow oil.

^1^H NMR (CDCl_3_) δ: 4.97 (m, 2H, NCH_2_), 5.25 (m, 2H, = CH_2_), 6.05 (m, 1H, CH), 6.76 (dd, *J* = 7.8 Hz, *J* = 4.8 Hz, 2H, H_3_, H_7_), 7.19 (dd, *J* = 7.8 Hz, *J* = 1.8 Hz, 2H, H_4_, H_6_), 8.01 (dd, *J* = 4.8 Hz, *J* = 1.8 Hz, 2H, H_2_, H_8_). EI MS *m*/*z*: 241 (M, 55), 200 (M-CH_2_CHCH_2_, 100). HR MS (EI) *m*/*z* calc. for [C_13_H_11_N_3_S + H] 242.0752. Found 242.0758.

10-Benzyl-1,9-diazaphenothiazine (**5**) (0.046 g, 63%); an beige oil.

^1^H NMR (CDCl_3_) δ: 5.64 (s, 2H, CH_2_), 6.73 (dd, *J* = 7.8 Hz, *J* = 4.8 Hz, 2H, H_3_, H_7_), 7. 26 (m, 7H, H_4_, H_6_, C_6_H_5_), 7.95 (dd, *J* = 4.8 Hz, *J* = 1.8 Hz, 2H, H_2_, H_8_). EI MS *m*/*z*: 291 (M, 100), 200 (M-CH_2_C_6_H_5_, 80). HR MS (EI) *m*/*z* calc. for [C_17_H_13_N_3_S + H] 292.0908. Found 292.0925.

#### Synthesis of 10-propargyl-1,9-diazaphenothiazines (6)

To a suspension of 10*H*-1,9-diazaphenothiazine (**2**) (0,100 g, 0.5 mmol) in dry DMF (10 ml) was added 80 mg (0.72 mmol) potassium *tert*-butoxide. The mixture was stirred at room temperature for 1 h. Then to the solution was added drop-wise a solution of propargyl bromide (0.080 g, 0.64 mmol) in dry toluene. The solution stirred at room temperature 24 h and poured into water (20 ml), extracted with methylene chloride (20 ml), dried with Na_2_SO_4_, evaporated to the beige oil. The residue was purified by column chromatography (silica gel, CHCl_3_) to yield 10-propargyl-1,9-diazaphenothiazine (**6**) (0.085 g, 71%); mp 119–120 °C.

^1^H NMR δ: 2.17 (s, 1H, CH), 5.07 (s, 2H, CH_2_), 6.84 (dd, *J* = 7.5 Hz, *J* = 5.1 Hz, 2H, H_3_, H_7_), 7.28 (dd, *J* = 7.8 Hz, *J* = 1.8 Hz, 2H, H_4_, H_6_), 8.12 (dd, *J* = 4.8 Hz, *J* = 1.8 Hz, 2H, H_2_, H_8_). EI MS: 239 (M, 90), 200 (M-CH_2_CCH, 100). HR MS (EI) *m*/*z* calc. for [C_13_H_9_N_3_S + H] 240.0595. Found 240.0599.

#### Synthesis of 10-substituted 1,9-diazaphenothiazines (7–12)

To a solution of 10*H*-1,9-diazaphenothiazine (**2**) (0.100 g, 0.5 mmol) in dry dioxane (10 ml) NaOH (0.200 g, 5 mmol) was added. The mixture was refluxed for 2 h and hydrochlorides of dialkylaminoalkyl chloride (3-dimethylaminopropyl, 2-diethylaminoethyl, 3-dimethylamino-2-methylpropyl) and hydrochlorides of cycloaminoethyl chloride [1–(2-chloroethyl)-pyrrolidine, 1–(2-chloroethyl)piperidine, 2–(2-chloroethyl)-1-methylpiperidine, 1.5 mmol] were added. The reaction mixture was refluxed for 48 h. After cooling dioxane was evaporated *in vacuo* and residue was dissolved in CHCl_3_ (10 ml). The extracts were washed with water, dried with anhydrous sodium sulphate and evaporated *in vacuo*. The obtained product was purified by column chromatography (aluminium oxide, CH_2_Cl_2_) to give the following:

10–(3′-Dimethylaminopropyl)-1,9-diazaphenothiazine (**7**) (0.100 g, 71%); an oil.

^1^H NMR δ: 1.97 (m, 2H, CH_2_), 2.24 (s, 6H, 2CH_3_), 2.44 (t, *J* = 7.5 Hz, 2H, NCH_2_), 4.22 (t, *J* = 7.5 Hz, 2H, NCH_2_), 6.84 (dd, *J* = 7.5 Hz, *J* = 5.1 Hz, 2H, H_3_, H_7_), 7.51 (dd, *J* = 7.8 Hz, *J* = 1.8 Hz, 2H, H_4_, H_6_), 8.33 (dd, *J* = 4.8 Hz, *J* = 1.8 Hz, 2H, H_2_, H_8_). CI MS *m*/*z*: 287 (M + 1, 100). HR MS (EI) *m*/*z* calc. for [C_15_H_18_N_4_S + H] 287.1330. Found 287.1326.

10–(2′-Diethylaminoethyl)-1,9-diazaphenothiazine (**8**) (0.105 g, 69%); an oil.

^1^H NMR δ: 1.08 (t, *J* = 7.2 Hz, 6H, 2CH_3_), 2.64 (q, *J* = 7.2 Hz, 4H, 2CH_2_), 2.80 (t, *J* = 7.2 Hz, 2H, NCH_2_), 4.03 (t, *J* = 7.2 Hz, 2H, NCH_2_), 6.85 (dd, *J* = 7.5 Hz, *J* = 5.1 Hz, 2H, H_3_, H_7_), 7.51 (dd, *J* = 7.8 Hz, *J* = 1.8 Hz, 2H, H_4_, H_6_), 8.31 (dd, *J* = 4.8 Hz, *J* = 1.8 Hz, 2H, H_2_, H_8_). CI MS *m*/*z*: 301 (M + 1, 20), 228 (M + 1-NC_4_H_10_, 100), 200 (M + 1-C_2_H_4_NC_4_H_10_, 25). HR MS (EI) *m*/*z* calc. for [C_16_H_20_N_4_S + H] 301.1486. Found 301.1476.

10–(3′-Dimethylamino-2′-methylpropyl)-1,9-diazaphenothiazine (**9**) (0.115 g, 80%); an oil.

^1^H NMR (CDCl_3_) δ: 0.92 (d, *J* = 6.5 Hz, 3H, CH_3_), 2.39 (m, 9H, 2CH_3,_ NCH_2_, CH), 4.15 (m, 2H, NCH_2_), 6.85 (dd, *J* = 7.5 Hz, *J* = 5.1 Hz, 2H, H_3_, H_7_), 7.51 (dd, *J* = 7.8 Hz, *J* = 1.8 Hz, 2H, H_4_, H_6_), 8.33 (dd, *J* = 4.8 Hz, *J* = 1.8 Hz, 2H, H_2_, H_8_). FAB MS *m*/*z*: 301 (M + 1, 100), 202 (M + 1-C_2_H_4_NC_4_H_10_, 20). HR MS (EI) *m*/*z* calc. for [C_16_H_20_N_4_S + H] 301.1487. Found 301.1494.

10–(2′-Pyrrolidinylethyl)-1,9-diazaphenothiazine (**10**) (0.110 g, 75%); an oil.

^1^H NMR (CDCl_3_) δ: 1.87 (m, 4H, 2CH_2_), 2.83 (m, 4H, 2NCH_2_), 2.99 (t, *J* = 7.5 Hz, 2H, NCH_2_), 4.44 (t, *J* = 7.5 Hz, 2H, NCH_2_), 6.85 (dd, *J* = 7.5 Hz, *J* = 5.1 Hz 2H, H_3_, H_7_), 7.51 (dd, *J* = 7.8 Hz, *J* = 1.8 Hz, 2H, H_4_, H_6_), 8.33 (dd, *J* = 4.8 Hz, *J* = 1.8 Hz, 2H, H_2_, H_8_). CI MS *m*/*z*: 299 (M + 1, 40), 202 (M + 1-C_2_H_4_NC_4_H_8_, 100). HR MS (EI) *m*/*z* calc. for [C_16_H_20_N_4_S + H] calc. 299.1330. Found 299.1324.

10–(2′-Piperydinylethyl)-1,9-diazaphenothiazine (**11**) (0.110 g, 70%); an oil.

^1^H NMR (CDCl_3_) δ: 1.48 (m, 2H, CH_2_),1.61 (m, 4H, 2CH_2_) 2.52 (m, 4H, 2NCH_2_), 2.68 (t, *J* = 6.8 Hz, 2H, NCH_2_), 4.13 (t, *J* = 6.8 Hz, 2H, NCH_2_), 6.84 (dd, *J* = 7.5 Hz, *J* = 5.1 Hz 2H, H_3_, H_7_), 7.51 (dd, *J* = 7.8 Hz, *J* = 1.8 Hz; 2H, H_4_, H_6_), 8.33 (dd, *J* = 4.8 Hz; *J* = 1.8 Hz, 2H, H_2_, H_8_). CI MS *m*/*z*: 313 (M + 1, 100), 202 (M + 1-C_2_H_4_NC_5_H_10_, 20). HR MS (EI) *m*/*z* calc. for [C_17_H_20_N_4_S + H] 313.1486. Found 313.1483.

10–(1′-Methyl-2′-piperidinylethyl)-1,9-diazaphenothiazine (**12**) (0.114 g, 71%); an oil.

^1^H NMR (CDCl_3_) δ: 2.10 (m, 7H), 2.38 (s, 3H, CH_3_), 2.94 (m, 1H, NCH), 4.02 (m, 2H, NCH_2_), 6.85 (dd, *J* = 7.5 Hz, *J* = 5.1 Hz 2H, H_3_, H_7_), 7.51 (dd, *J* = 7.8 Hz, *J* = 1.8 Hz; 2H, H_4_, H_6_), 8.33 (dd, *J* = 4.8 Hz; *J* = 1.8 Hz, 2H, H_2_, H_8_). CI MS *m*/*z*: 327 (M + H, 80), 313 (M + 1-CH_3_ 100). HR MS (EI) *m*/*z* calc. for [C_18_H_22_N_4_S + H] 327.1643. Found 327.1639.

### Crystal data

10-methyl-1,9-diazaphenothiazine (**3**), *M* = 215.27, yellow needle, 0.23 × 0.09 × 0.06 mm, monoclinic *P*2_1_/*c* space group, *V* = 941.73(5) Å^3^, *Z* = 4, *D*_c_ = 1.518 g/cm^3^, *F*_000_ = 448, SuperNova Dual, Cu*Kα* radiation, *λ* = 1.54184 Å, *T* = 100(2) K, 2*Θ*_max_ = 70.142°, 16,026 reflections collected, 1773 unique (*R*_int_ = 0.080). Final *GooF* = 1.043, *R* = 0.035, *wR* = 0.090, *R* indices based on 1725 reflections with *I* > 2*σ*(*I*) (refinement on *F*^2^), 137 parameters, 0 restraints. Lp and absorption corrections applied, *µ* = 2.754 mm^−1^. CCDC 1865083.

### Biological evaluation

#### Cell culture

Compounds were evaluated for their anticancer activity using three cultured cell lines: SNB-19 (human glioblastoma, DSMZ – German Collection of Microorganisms and Cell Cultures, Braunschweig, Germany), C-32 (human amelanotic melanoma, ATCC-American Type Culture Collection, Manassas, VA), MDA-MB-231 (human adenocarcinoma mammary gland, ATCC, Manassas, VA) and HFF-1 (human fibroblast cell line, ATCC, Manassas, VA). The cultured cells were kept at 37 °C and 5% CO_2_. The cells were seeded (1 × 10^4^ cells/well/100 µl DMEM supplemented with 10% FCS and streptomycin and penicillin) using 96-well plates (Corning). The cells were counted in a haemocytometer (Burker’s chamber) using a phase contrast Olympus IX50 microscope equipped with Sony SSC-DC58 AP camera and Olympus DP10 digital camera.

#### Proliferation assay

The antiproliferative effect of the compounds obtained from both the cancer and the normal cells was determined using the Cell Proliferation Reagent WST-1 assay (Roche Diagnostics, Mannheim, Germany). This colorimetric assay is based on the viable cell’s ability to cause the bright red-coloured stable tetrazolium salt (2–(4-iodophenyl)-3–(4-nitrophenyl)-5–(2,4-disulfophenyl)-2H-tetrazolium, monosodium salt) to cleave to the dark red soluble formazan by cellular enzymes. An expansion in the number of viable cells results in an increase in the overall activity of mitochondrial dehydrogenases in the sample. An increase in the amount of formazan dye formed correlates to the number of metabolically active cells in the culture. The formazan dye produced by metabolically active cells is quantified by a scanning ELISA reader that measures the absorbance of the dye solution at appropriate wavelengths. The examined cells were exposed to the tested compounds for 72 h at various concentrations between 0.1 and 100 µg/ml (prepared initially at a concentration of 1 mg/ml in DMSO). The control was performed in order to check that DMSO has no effect on the cells at the concentration used. The cells were incubated with WST-1 (10 µl) for one hour and the absorbance of the samples was measured against a background control at 450 nm using a microplate reader with a reference wavelength at 600 nm. The results are expressed as the means of at least two independent experiments performed in triplicate. The antiproliferative activity of the tested compound was compared to cisplatin. The IC_50_ values (a concentration of a compound that is required for 50% inhibition) were calculated from the dose–response relationship with respect to control.

#### The RT-QPCR method

Genes trancriptional activity (*H3*, *TP53*, *CDKN1A*, *BCL-2*, *BAX*) was evaluated by the real-time RT-QPCR method with OPTICON TM DNA Engine (MJ Research, Watertown, MA) and QuantTect^®^ SYBR^®^ Green RT-PCR Kit (Quiagen, Valencia, CA). Cells were exposed to compounds **5** and **8** at a concentration of 0.5 µg/ml for 24 h. The RNA extraction was made by using Quick-RNA™ Kit MiniPrep (ZYMO RESEARCH). Total RNA integrity was analysed in 1.2% agarose electrophoresis with added ethidium bromide compound. The quantity and purity of extracted total RNA were determined by using spectrophotometric analysis with HP845 (Hewlett Packard, Waldbronn, Germany) spectrophotometer. The statistical analysis was performed using the Statistica 8.0 software (StatSoft, Tulsa, OK). All values were expressed as means ± SE.

#### Apoptosis antibody detection array

The Proteome Profiler Human Apoptosis Array (R&D Systems) kit simultaneously detects the relative expression level of 35 apoptosis-related proteins. In short, the SNB-19 cells were treated with compounds **5** and **9** at a concentration of 0.5 µg/ml. All immunodetection steps were performed in accordance with the manufacturer’s instruction. The blots were detected using an enhanced chemiluminescence system using LI-COR C-Digit Blot Scanner.

#### Annexin-V apoptosis detection assay

Apoptosis was analysed via Annexin-V-FLUOS Staining kit (Roche, Germany) according to the manufacture’s instruction. Briefly, after 24 h incubation of the SNB-19 cells with compounds **5** and **8** (0.5 µg/ml), the samples were collected, washed with phosphate-buffered solution and treated with annexin and propidium iodide. The stained cells were analysed by a flow cytometry (LSR II Becton Dickinson flow cytometer). Viable, early apoptopic, late apoptopic and necrotic cells were determined by staining Annexin V^−^/PI^−^, Annexin V^+^/PI^−^, Annexin V^+^/PI^+^ Annexin V^−^/PI^+^, respectively.

## Results and discussion

### Synthesis

Synthesis of known dipyridothiazines is dependent on the location of the nitrogen atoms in the formed products. Synthesis from aminonitrodipyridinyl (2,2′-, 2,4′- and 4,4′-) sulphides or from two appropriate disubstituted pyridines (proceeding through a sulphide formation step without isolation) runs as the Smiles rearrangement to dipyridinylamine (usually not isolated) followed by cyclisation to form the 1,4-thiazine ring. In this way, four dipyridothiazines were obtained (1,6-diaza-^34^^,^^41–45^, 1,8-diaza-^33^^,^^46^, 2,7-diaza-^47^^,^^48^ and 3,6-diazaphenothiazines^35^^,^^49^. Direct cyclisation as the Ullmann-type process to 2,6-diaza-, 2,8-diaza- and 4,6-diazaphenothiazines has not been observed^50^. Symmetrical 1,9-diaza- and 3,7-diazaphenothiazines were obtained by sulphurisation of 2,2′- and 4,4′-dipyridinylamine with elemental sulphur at higher temperature. The synthesis of 10*H*-1,9-diazaphenothiazine (**2**) was mentioned by Rath^40^ as a procedure of preparation being similar to the process used for converting diphenylamine to phenothiazine (without any details of the reaction conditions, and the product isolation and characterisation). The synthesis of isomeric 10*H*-3,7-diazaphenothiazine in the reaction of sulphurisation of 4,4′-dipyridinylamine was performed at 280–290 °C giving the product in very yield (9%)^51^.

Our sulphurisation of 2,2′-dipyridinylamine (**1**) with elemental sulphur at 250 °C for 1 h in open air conditions (without a solvent) gave 10*H*-1,9-diazaphenothiazine (**2**) in 28% yield. Sulphurisation in an autoclave using dioxane as a solvent was less efficient than in open air giving the desired product in 17% yield. Atempted sulphurisations in other solvents (ethylene glycol in open air conditions and water, chloroform, DMF and monomethyl ether of diethylene glycol in an autoclave) were not satisfactory. The best result (42%) was achieved when the reaction (without a solvent) was carried in a microwave reactor.

**Scheme 1.**
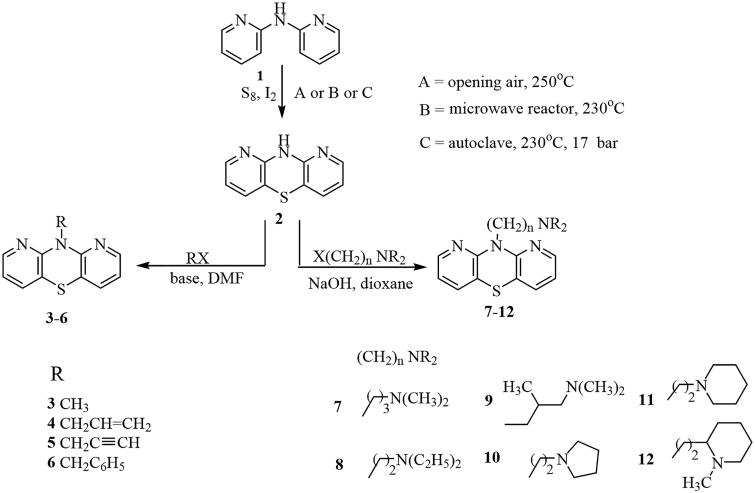
Synthesis of 10H-1,9-diazaphenothiazine (**2**) and its 10-derivatives (**3–12**).

10*H*-1,9-diazaphenothiazine (**2**) was transformed into 10-substituted derivatives **3–12** in alkylation reaction with selected alkyl and dialkylaminoalkyl halides (Scheme 1).

### Spectroscopic analysis

The Rath product was not characterised at all^40^. Our spectroscopic study revealed a molecular formula of C_10_H_7_N_3_S from HR MS spectrum and only 3 pyridine proton signals of an AMX system in the ^1^H NMR spectrum which pointed at a symmetrical structure of the sulphurisation product. To find the location of the azine nitrogen atoms in the dipyridothiazine system, the sulphurisation product was methylated with methyl iodide in dry DMF in the presence of sodium hydride. The simple ^1^H NMR spectrum revealed four signals: the methyl group and three pyridine protons confirming that the methyl group is attached to the thiazine nitrogen atom.

To find the pyridine nitrogen atoms 2D NMR ROESY spectrum was recorded. An irradiation of the methyl protons at 3.45 ppm did not show any proximity of the methyl group to the protons confirming the pyridine nitrogen atoms to be in positions 1 and 9. The full assignment of the proton and carbon signals came from other 2D NMR spectra showing ^1^H-^1^H (COSY) and ^13^C-^1^H connectivities (HSQC and HMBC, in Supplementary Material). Those last two spectra showed the C-H relationship through one bond (^1^*J*_C,H_ connectivity) and three bonds (^3^*J*_C,H_ connectivity).

The all ^1^H-^1^H and ^1^H-^13^C relationships were included in Supplementary Material. This spectroscopic analysis confirmed the methylated product to be 10-methyl-1,9-diazaphenothiazine (10-methyldipyrido[3,2-b;2′,3′-e]^1^^,^^4^ thiazine) (**3**).

### X-ray diffraction study

Such a high temperature process could lead to many stable and unstable compounds and some rearrangements cannot be neglected. The NMR analysis is an indirect and subtle method of the structure elucidation, a single-crystal X-ray diffraction analysis of 10-methyl derivative (**3**) was performed (the direct sulphurisation product did not give crystals good enough for X-ray diffraction measurement).

**Figure 1.**
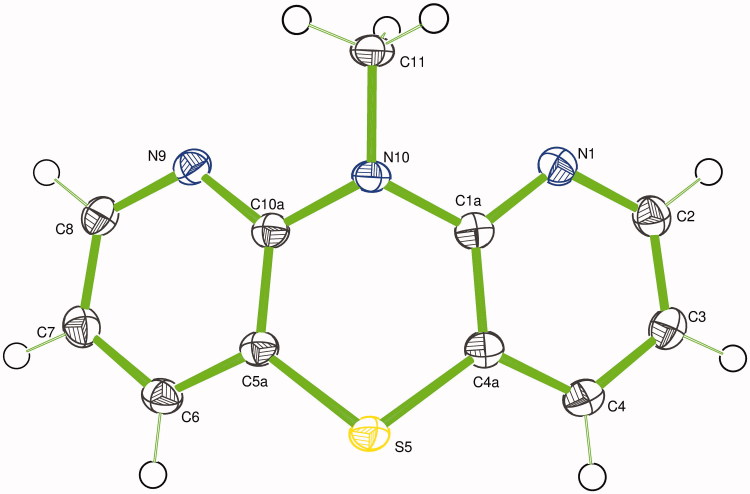
Ortep drawing of 10-methyl-1,9-diazaphenothiazine (**3**), showing the atom labelling.

The X-ray diffraction study confirmed the product structure concluded from the ^1^H NMR spectra as 10-methyl-1,9-diazaphenothiazine and revealed a spatial arrangement in the molecule in a solid state (Figure 1).

In all known studies of *N*-methyl dipyridothiazine crystals, the tricyclic ring systems are planar or folded depending on the methyl group location^35^^,^^47^^,^^52^. The ring system in compound (**3**) is also folded along the S–N axis with the butterfly angle of 36.22(4)° between two pyridine ring planes. The central thiazine ring is in boat conformation with the angle between two halves (SCCS) of 41.28(6)°. The methyl group is located in equatorial position with the S5···N10–C11 angle of 170.1(1)°.

The bond angles on heteroatoms in the central ring C1a–N10–C10a and C4a–S5–C5a are 118.74(12)° and 98.19(7)°, respectively. The thiazine nitrogen atom (N10) showed pyramidal configuration as a sum of three bond angles around this atom is 353.12°. The N10–C bond lengths in the thiazine ring are 1.406–1.409 Å, being significantly longer than the azine N–C bonds (1.331–1.349 Å). The N10–CH_3_ bond length is the longest at 1.463(2) Å due to the sp^3^ hybridisation of the carbon atom. The molecule interacts with the neighbouring four molecules with four non-classical C–H…N hydrogen bonds (the molecule is an acceptor of two and a donor of two others H-bonds) forming 2D layers perpendicular to the *c* crystallographic axis. Consequently, a layer-type structure was observed.

### Anticancer activity

As it was mentioned in Introduction, some 1,6-diaza-, 1,8-diaza-, 2,7-diaza- and 3,6-diazaphenothiazines substituted at the thiazine nitrogen atom, exhibited very strong anticancer action against various cancer cell lines. The most active compounds possessed the propargyl and dialkylaminoalkyl substituents, and in some cases even the hydrogen atom^33–35^^,^^38^^,^^39^. Therefore, 10*H*-1,9-diazaphenothiazine (**2**) and its 10-derivatives **3–12** with various alkyl and dialkylaminoalkyl substituents were screened for their anticancer activity against *in vitro* using cultured glioblastoma SNB-19, melanoma C-32 and breast cancer MDA-MB-231 cell lines and cisplatin as a reference drug. Besides of the dimethylaminomethylpropyl derivative **9** all the compounds turned out to be very active at least one cancer cell line (Table 1). This type of dipyridothiazine appeared the most potent comparing with other anticancer tested dipyridothiazines (1,6-, 1,8-, 2,7- and 3,6-). The parent compounds (**2**) was very active against melanoma C-32 (IC_50_ = 3.83 µM, more potent than cisplatin, IC_50_ = 13,2 µM) and inactive against other lines. General, an introduction of alkyl and dialkylaminoalkyl groups in position 10 enhanced the activity. Compounds **5–8** and **11** with the propargyl, benzyl, dimethylaminopropyl, diethylaminoethyl and morpholinylethyl groups were highly active against glioblastoma SNB-19 with IC_50_ = 0.33–3.85 µM. Compounds **6–8** and **10** (having the pyrrolidinylethyl group) was very active against breast cancer MDA-MB-231 with IC_50_ = 2.13–9.61 µM. Compounds **3–5** (possessing the methyl, allyl and propargyl groups) exerted strong activity against melanoma C-32 with IC_50_ = 3.37–4.02 µM. The most active compounds were more potent than cisplatin.

**Table 1.** The anticancer activity of 1,9-diazaphenothiazines.

No	Anticancer activity IC_50_ (μμ)		
	SNB-19	C-32	MDA-MB-231
**2**	>200	3.83	>200
**3**	>200	3.72	29.5
**4**	172.2	4.02	19.1
**5**	3.85	3.37	14.1
**6**	0.79	28.2	7.36
**7**	0.38	50	9.61
**8**	0.33	14.4	2.13
**9**	>200	>200	>200
**10**	>200	>200	8.52
**11**	3.62	21.5	14.2
**12**	>200	>200	29.8
***Cisplatin***	3.73	13.2	25.8

### Apoptosis assay

Compounds **5** and **8** were selected as the most promising 1,9-diazaphenothiazines to study the mechanism of anticancer action using the RT-QPCR method. This method analysed the gene transcriptional activities of proliferation marker (*H3*) cell cycle regulator (*TP53* and *CDKNIA*) and intracellular apoptosis pathway (*BACL-2* and *BAX*). The obtained results on three cancer cell lines are collected in Table 2.

**Table 2.** The influence of compounds **5** and **8** on expression of genes encoding *H3*, *TP53*, *CDKN1A*, *BCL-2*, *BAX*.

Gene	number of mRNA copies/μg total RNA		
	SNB-19	C-32	MDA-MB-231
*H3*			
Control	17,909 ± 1812	995,904 ± 181,187	183,104 ± 32,058
** 5**	3717 ± 408	18,538 ± 584	6374 ± 609
** 8**	4448 ± 420	366,840 ± 33,506	44,374 ± 2912
*TP53*			
Control	835,734 ± 53,484	682,740 ± 84,734	299,833 ± 4213
** 5**	1,398,619 ± 245,646	500,868 ± 23,049	110,292 ± 29,641
** 8**	503,813 ± 66,078	636,089 ± 36,620	194,767 ± 54,249
*CDKN1A*			
Control	242,104 ± 131,105	1,752,117 ± 374,944	17,642 ± 2504
** 5**	442,447 ± 43,883	1,724,825 ± 162,708	100,681 ± 10,814
** 8**	348,748 ± 50,156	2,244,254 ± 156,054	83,652 ± 4606
*BCL-2*			
Control	15,315 ± 1085	68,431 ± 12,161	121,241 ± 13,879
** 5**	16,710 ± 622	23,277 ± 6412	29,376 ± 3256
** 8**	12,765 ± 2127	74,397 ± 7567	88,717 ± 11,951
*BAX*			
Control	918,016 ± 47,034	2,431,793 ± 574,891	154,191 ± 11,428
** 5**	1,090,855 ± 133,864	490,262 ± 29,522	121,389 ± 12,855
** 8**	1,198,420 ± 338,142	1,855,193 ± 488,197	181,425 ± 27,675
*BAX/BCL-2*			
Control	59.9	35.5	1.21
** 5**	65.3	21.1	4.13
** 8**	93.9	24.9	2.04

The growth, division and eventual death of the cells in the body are processes that are controlled by hundreds of genes working together. The gene encoding the histone *H3* is involved in the cell cycle progression and is considered as an indicator of proliferation in molecular studies. It plays an important role in regulation of the expression of the genetic information encoded in DNA^53^^,^^54^. Both compounds reduced considerably (**5** is more potent) the number of mRNA copies in all cancer lines what can be a result of a modification of the chromatin structure.

Tumour protein p53 is *TP53* gene product, one of the most known tumour suppressor genes, which is involved in anticancer action by various mechanisms. The p53 protein is activated by a variety of cell stresses, such as DNA damage, oncogene activation, spindle damage and hypoxia. Activated p53 transactivates a number of target genes, many of which are involved in DNA repair, cell cycle arrest and apoptosis^55–57^. Compounds **8** and **5** decreased mRNA copies in 3 or 2 cancer lines, respectively (only an increase in mRNA copies was observed in C-32 line).

The cell cycle inhibitor CDKN1A (p21) tightly controlled by the p53 protein is a protein playing multiple roles not only in the DNA damage response, but also in many cellular processes during unperturbed cell growth. The main and well-known function of protein p21 is to arrest cell cycle progression by inhibiting the activity of cyclin-dependent kinases. This protein is also involved in the regulation of transcription, apoptosis, DNA repair, as well as cell motility. As a biomarker of the cell response to different toxic stimuli, p21 expression and functions were analysed^58^^,^^59^. Compound **8** induced increase in the expression of *CDKN1A* in all cancer lines but compound **5** only in breast cancer lines.

P53 protein is also responsible for keeping the right balance between expression of a proapoptotic *BAX* gene and an antiapoptotic *BCL-2* gene. These proteins have special significance since they can determine if the cell commits to apoptosis or aborts the process. It is thought that the main mechanism of action of the *BCL-2* family of proteins is the regulation of cytochrome *c* release from the mitochondria via alteration of mitochondrial membrane permeability^60^. In general, compounds **5** and **8** reduced (with some exception) the mRNA expression of *BCL-2*. In contrast to this, the compounds enhanced or reduced the expression of *BAX*. The ratio of *BAX/BCL-2* can determine whether cells will die via apoptosis or be protected from it. In comparison with the control, the *BAX/BCL-2* ratio was found to be greater for both compounds in relation to SNB-19 and MDA-MB-231 cell lines.

In summary, the analysis of the gene expression revealed that compounds **5** and **8** selectively reduced expression of *H3* and *TP53*, and enhanced expression of *CDKN1A* in examined cell lines. The gene expression ratio of *BAX/BCL-2* indicated the induction of mitochondrial apoptosis in two cancer cell lines (SNB-19 and MDA-MB-231). In melanoma C-32 cell line, the transcriptional gene activity suggests a different way of cell death.

Annexin V Apoptosis Detection Assay showed the populations corresponding to viable, necrotic, early and late apoptopic cells. When the SNB-19 cells were treated with compounds **5** and **8**, there was slight increase in early and late apoptopic cell populations and slight decrease in viable cell populations (Supplementary Material).

To further understand the mechanism of action of compounds **5** and **8**, we performed a determination of apoptosis-related proteins using Proteome Profiler Human Apoptosis Array. We identified 12 expressed proteins in the response to the compounds in the SNB-19 cells (Supplementary Material). Proteins such as phospho-p53 (S15, phosphorylation at ser15), phospho-p53 (S46) and phospho-p53 (S392) play an important role in cell proliferation as DNA damage response, induction of apoptosis and growth suppression^61^ and all three proteins were found in the protein array. We found proteins which implicated in apoptosis: BAX, pro-caspase-3, cytochrome c and SMAC/Diablo. The last protein is a proapoptogenic mitochondrial protein which interacts and antagonises inhibitors of apoptosis proteins (IAPs) thus allowing the activation of caspases and apoptosis^62^. BAX accelerates programmed cell death by binding to mitochondrial membrane (MOMP) releasing cytochrome c, promoting activation of caspase-3 and triggering apoptosis^63^. It seems that compounds **5** and **8** induce BAX to form a channel in MOMP and release cytochrome c to activate caspases 9 and 3 (promoted also by SMAC/Diablo) thus initiating apoptosis through the intrinsic mitochondrial pathway. However, further studies are required to confirm the precise mechanism of this anticancer action.

## Conclusion

We report here efficient synthesis of 10*H*-1,9-diazaphenothiazine in the sulphurisation reaction of diphenylamine with elemental sulphur and its transformation into new 10-substituted derivatives, containing the alkyl and dialkylaminoalkyl groups at the thiazine nitrogen atom. The 1,9-diazaphenotiazine ring system was identified with advanced ^1^H and ^13^C NMR techniques and confirmed by single**-**crystal X-ray crystallography of the methyl derivative. X-ray diffraction analysis revealed nonplanar tricyclic ring system with the substituent at the thiazine nitrogen atom in an equatorial location. The compounds exhibited significant anticancer activities against the human glioblastoma SNB-19, melanoma C-32 and breast cancer MDA-MB231 cell lines. The most active 1,9-diazaphenothiazines were the derivatives with the propynyl and *N*, *N*-diethylaminoethyl groups being more potent than cisplatin. The expression of *H3*, *TP53*, *CDKN1A*, *BCL-2* and *BAX* genes for those two compounds was detected by the RT-QPCR method. The analysis of the gene expression revealed that both compounds inhibited the proliferation in all cells (*H3*) and activated of mitochondrial events of apoptosis (*BAX*/*BCL-2*) in two cancer cell lines (SNB-19 and MDA-MB-231). The proteome profiling study showed the most probable compound action on SNB-19 cells through the intrinsic mitochondrial pathway of apoptosis. The 1,9-diazaphenotiazine system seems to be more potent than known isomeric ones (1,6-diaza-, 1,8-diaza-, 2,7-diaza- and 3,6-diazaphenothiazine).

## Supplementary Material

Supplemental Material
