# Supplementary material for: 10H-1,9-diazaphenothiazine and its 10-derivatives: synthesis, characterisation and biological evaluation as potential anticancer agents
Source: J Enzyme Inhib Med Chem. 2019 Jul 16;34(1):1298–306. doi: 10.1080/14756366.2019.1639695 (PMC6691808; doi:10.1080/14756366.2019.1639695)

**Supplementary Material**

**10*H*-1,9-diazaphenothiazine and its 10-derivatives: synthesis, characterization and biological evaluation as potential anticancer agents**

**Beata Morak-Młodawska^1^, Krystian Pluta^1^*, Małgorzata Latocha^2^, Małgorzata Jeleń^1^, Dariusz Kuśmierz^2^, Kinga Suwińska^3,4^, Aleksander Shkurenko^5^, Zenon Czuba^6^, Magdalena Jurzak^2^**

^1^The Medical University of Silesia, School of Pharmacy with the Division of Laboratory Medicine, Department of Organic Chemistry, Jagiellońska 4, 41-200 Sosnowiec, Poland

^2^The Medical University of Silesia, School of Pharmacy with the Division of Laboratory Medicine, Department of Cell Biology, Jedności 8, 41-200 Sosnowiec, Poland

^3^Faculty of Mathematics and Natural Sciences, Cardinal Stefan Wyszyński University, K. Wóycickiego 1/3, 01-938 Warszawa, Poland

^4^A. M. Butlerov Institute of Chemistry, Kazan Federal University, Kremlevskaya ul. 18, Kazan, 420008, Russia

^5^Division of Physical Functional Materials Design, Discovery & Development Research Group (FMD3), Sciences and Engineering Advanced Membranes & Porous Materials (AMPM), King Abdullah University of Science and Technology (KAU ST), Thuwal, Kingdom of Saudi Arabia

^6^Department of Microbiology and Immunology, Medical University of Silesia in Katowice, Jordana 19, 41 808 Zabrze, Poland

**Content**

1. ^1^H NMR of 10*H*-1,9-diazaphenothiazine (**2**) 2
2. ^13^C NMR of 10*H*-1,9-diazaphenothiazine (**2**) 4
3. EI MS of 10*H*-1,9-diazaphenothiazine (**2**) 5
4. HR MS of 10*H*-1,9-diazaphenothiazine (**2**) 6
5. ^1^H NMR of 10-methyl-1,9-diazaphenothiazine (**3**) 7
6. ^13^C NMR of 10-methyl-1,9-diazaphenothiazine (**3**) 9
7. The COSY, HSQC and HMBC NMR experiments for 10-methyl-1,9-diazapheno-thiazine (**3**) 10
8. 2D NMR: COSY, ROESY, HSQC, HMBC of 10-methyl-1,9-diazaphenothiazine (**3**) 11
9. Table 1. The proton-proton and proton-carbon correlation in compound (**3**) (** **ppm) 15
10. EI MS of 10*H*-1,9-diazaphenothiazine (**3**) 16
11. HR MS of 10*H*-1,9-diazaphenothiazine (**3**) 17
12. A layer-type structure of 10-methyl-1,9-diazaphenothiazine (**3**) 18
13. Proteome Profiler™ - compounds (**5**) and (**8**) 19
14. The Flow Cytometry (FCM) of compounds (**5)** and (**8**) 19

1.^1^H NMR of 10*H*-1,9-diazaphenothiazine (**2**)

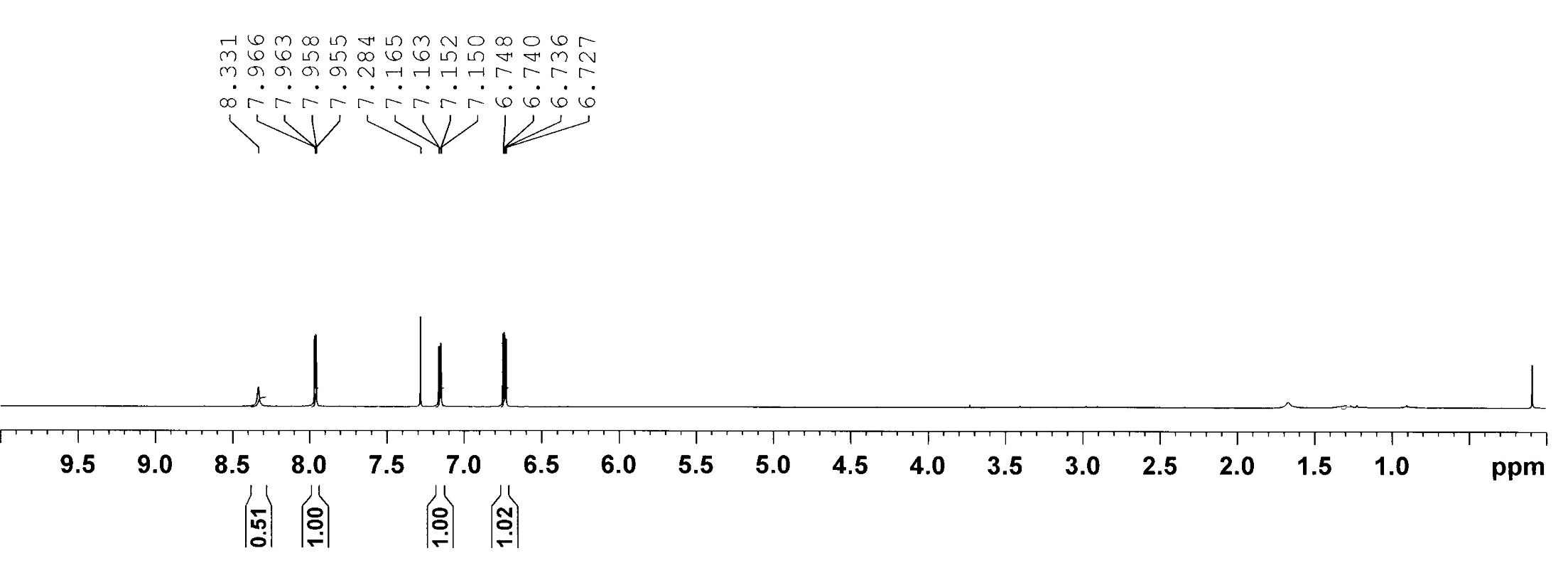


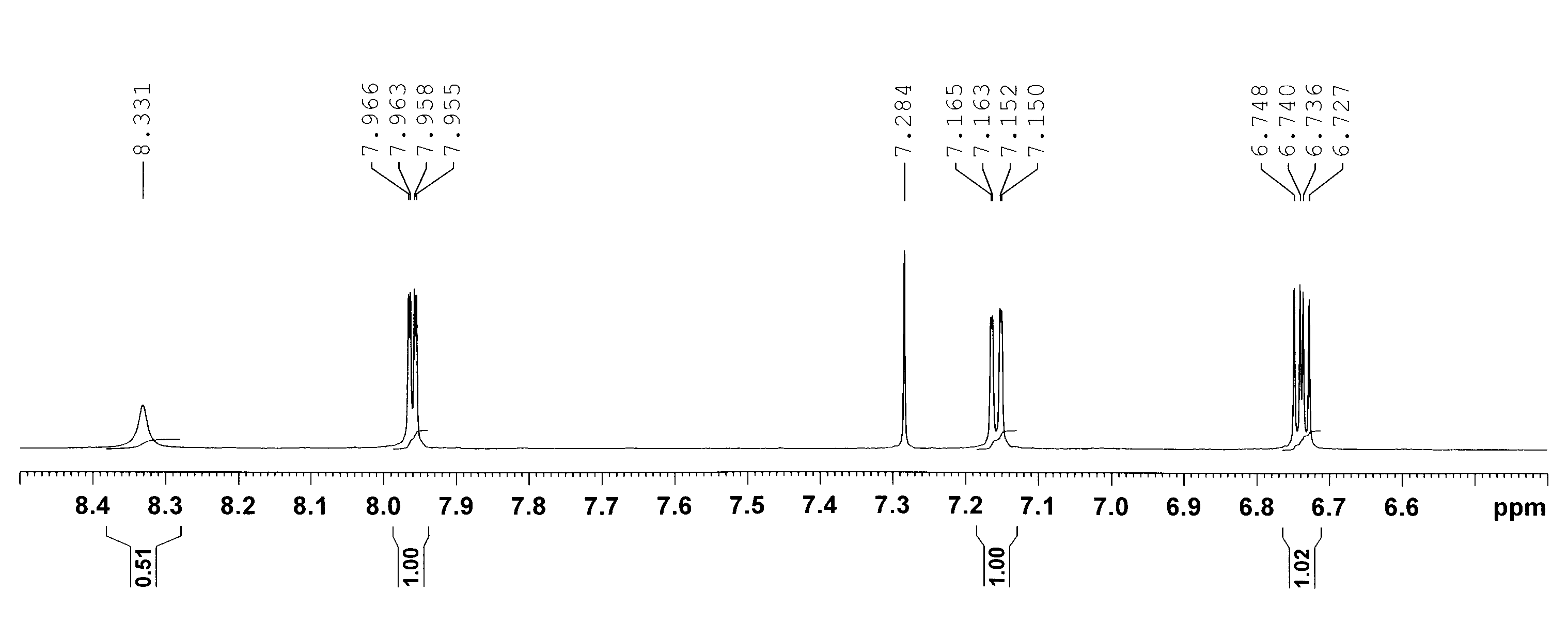


2. ^13^C NMR of 10*H*-1,9-diazaphenothiazine (**2**)


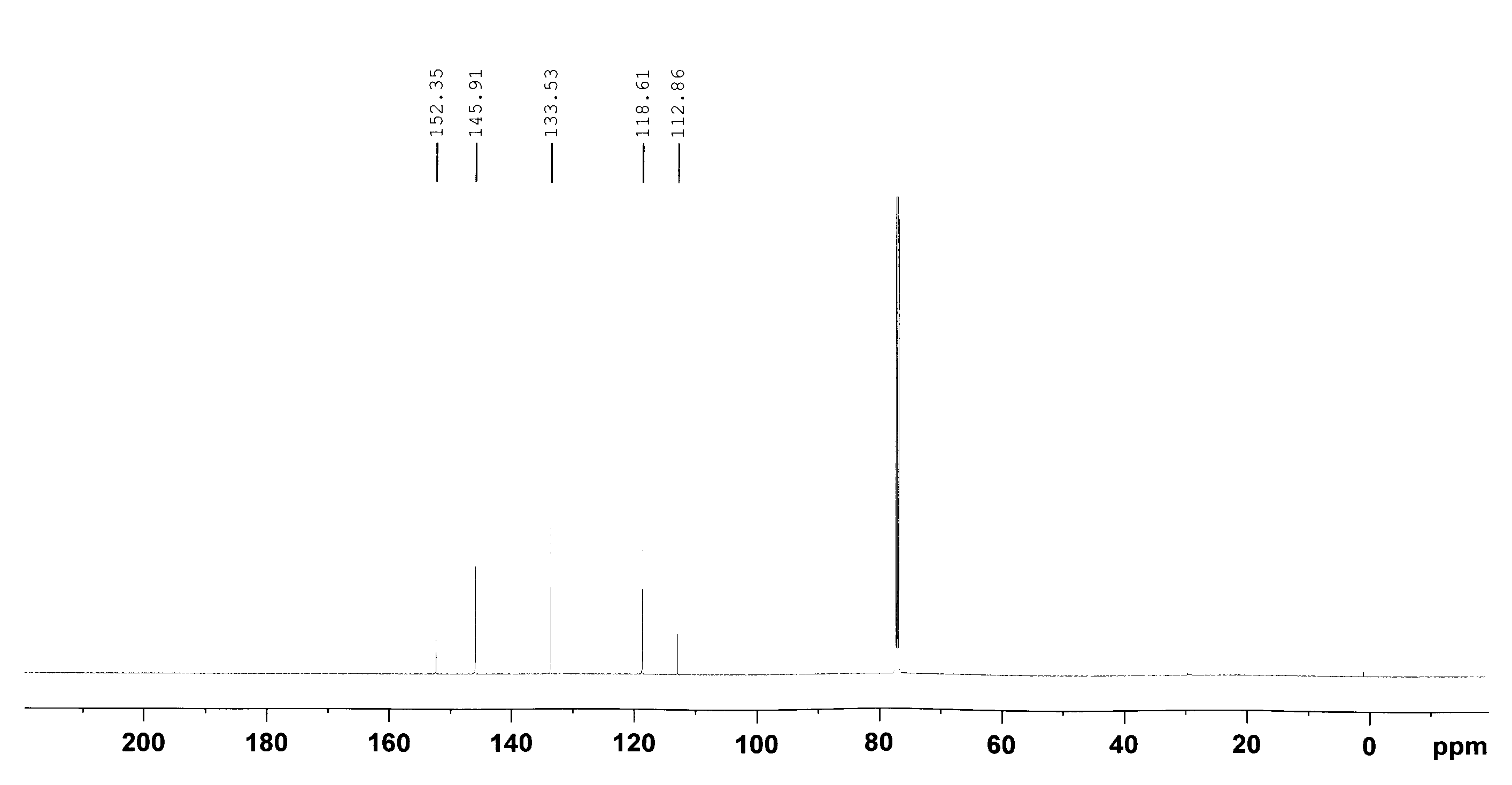


3. EI MS of 10*H*-1,9-diazaphenothiazine (**2**)


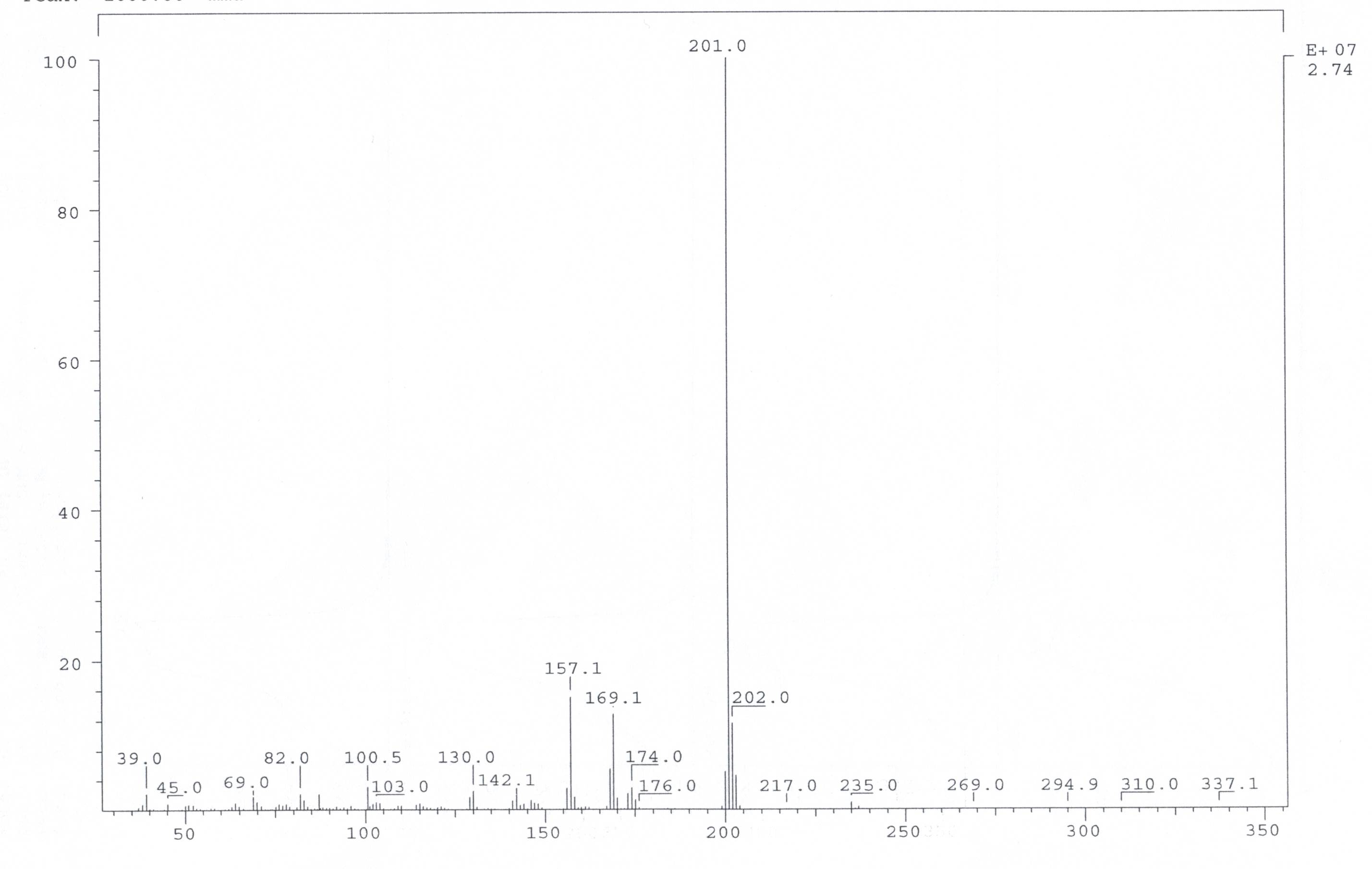


4. HR MS of 10*H*-1,9-diazaphenothiazine (**2**)


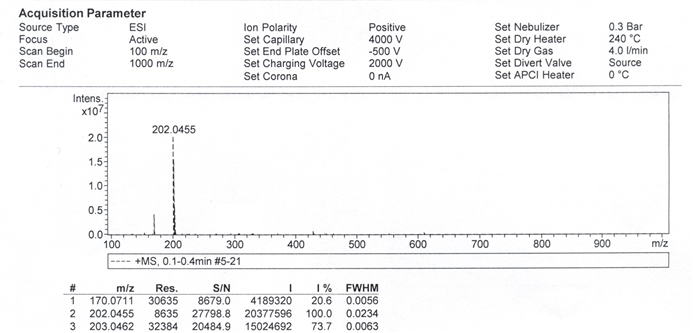


5. ^1^H NMR of 10-methyl-1,9-diazaphenothiazine (**3**)

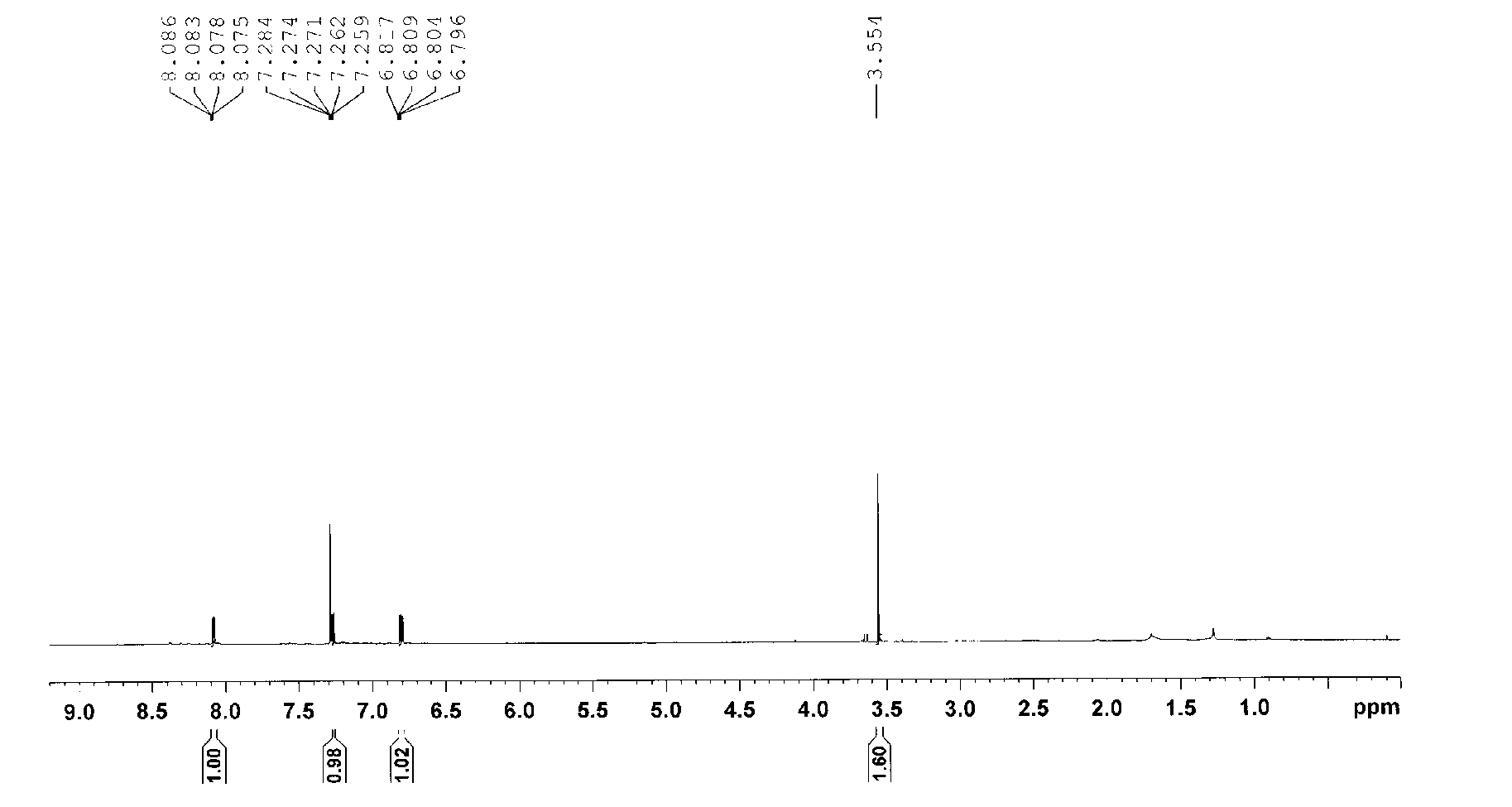


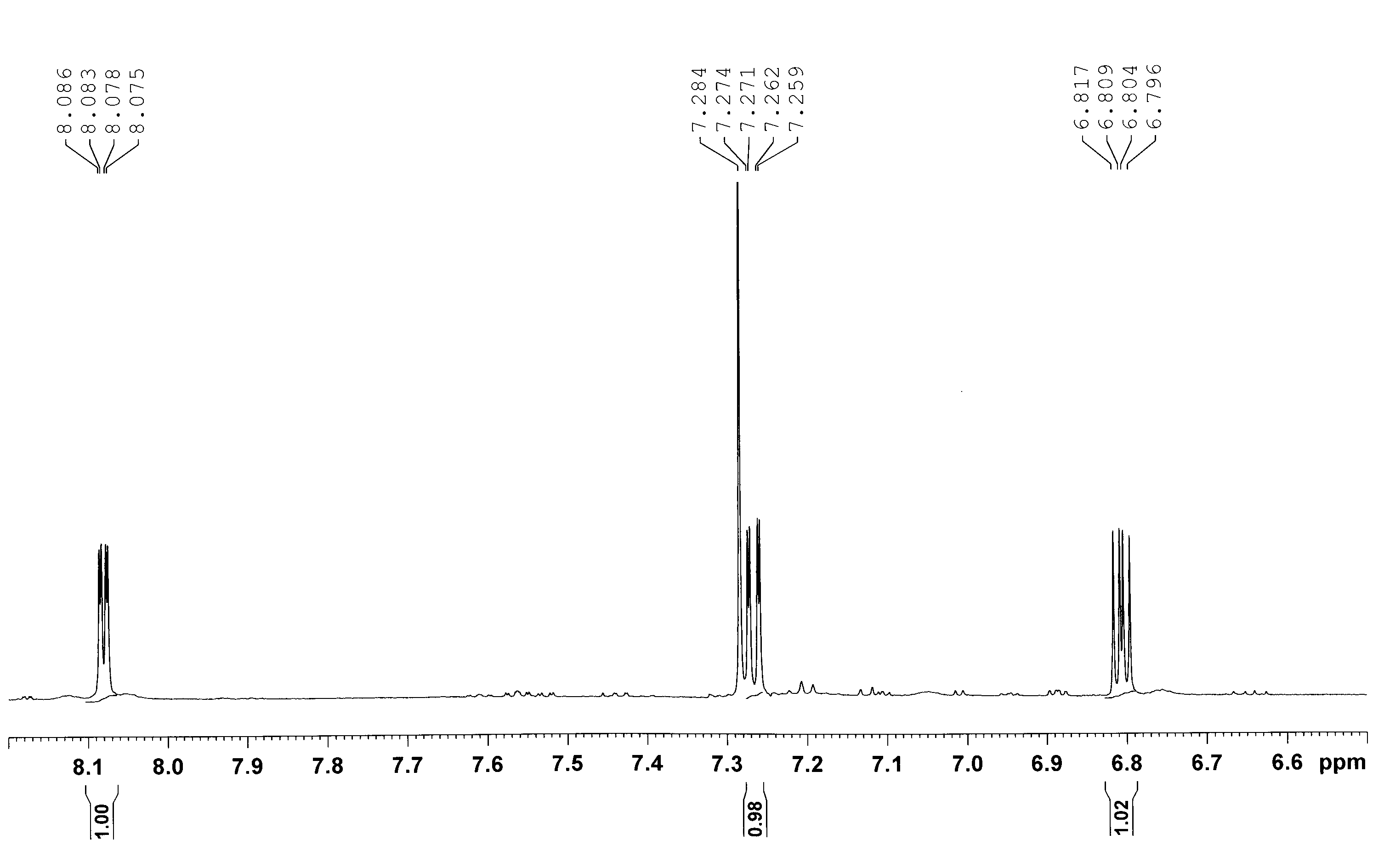


6. ^13^C NMR of 10-methyl-1,9-diazaphenothiazine (**3**)


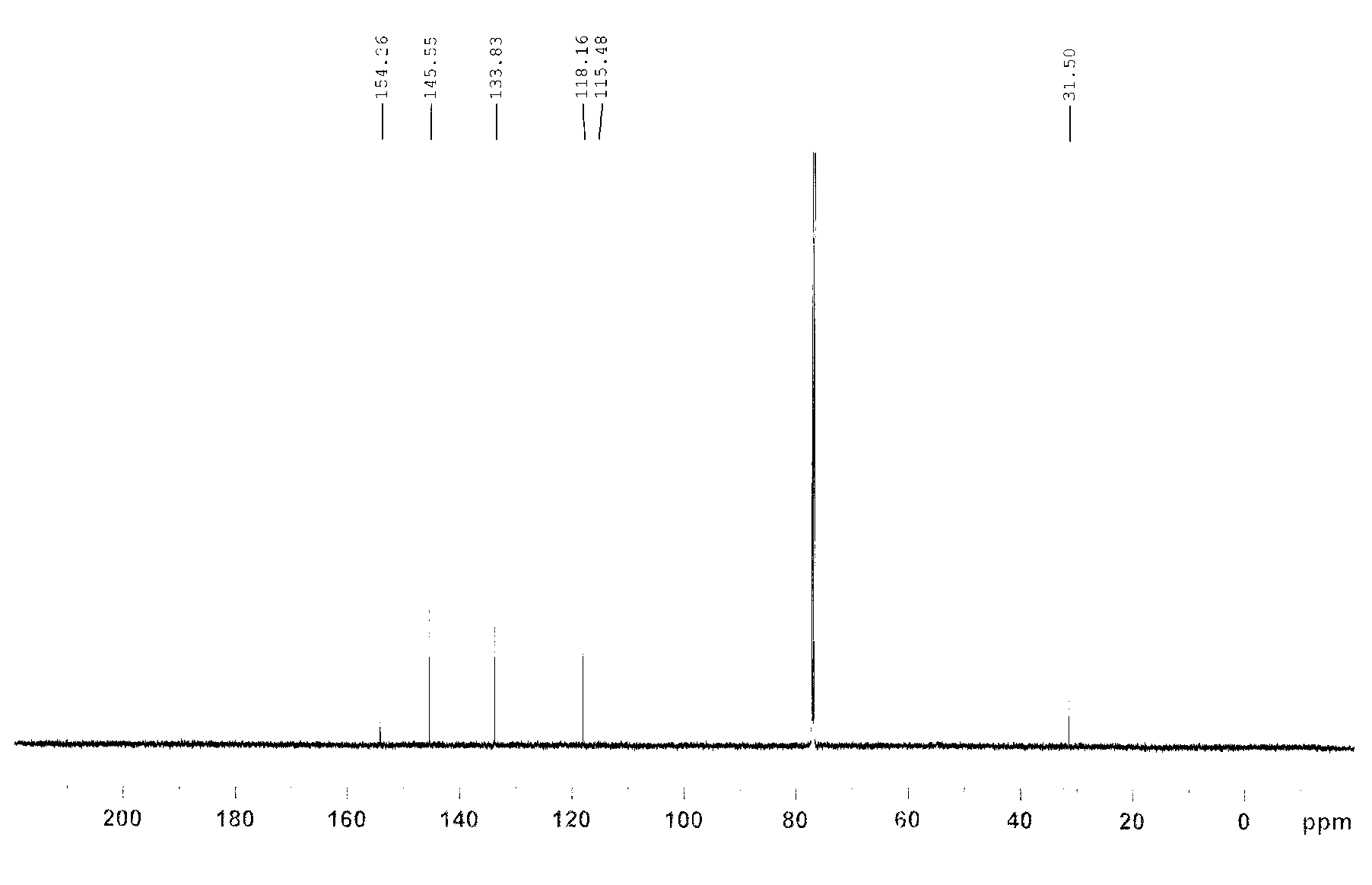


7. The COSY, HSQC and HMBC NMR experiments for 10-methyl-1,9-diazaphenothiazine (**3**).

8. 2D NMR: a. COSY, b. ROESY, c. HSQC, d. HMBC of 10-methyl-1,9-diazaphenothiazine (**3**)

a. ^1^H-^1^H COSY


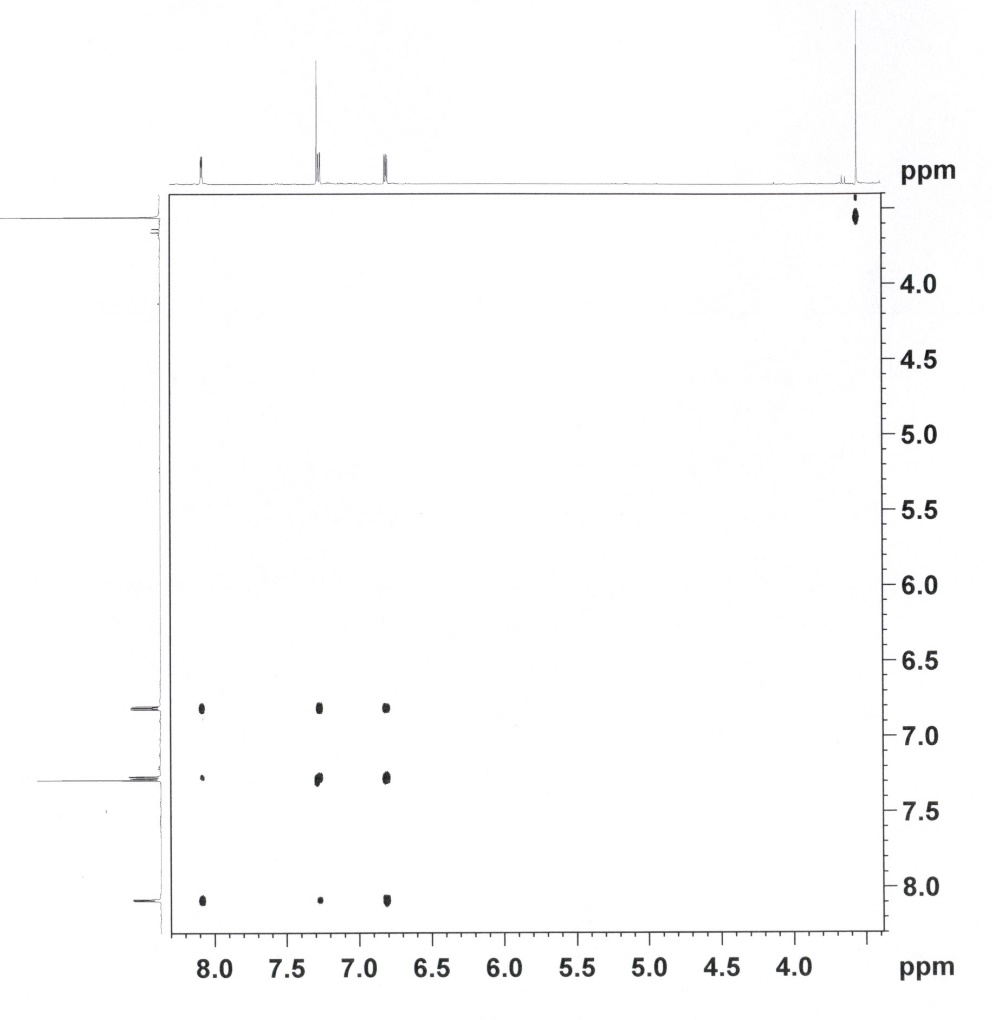


b. ROESY


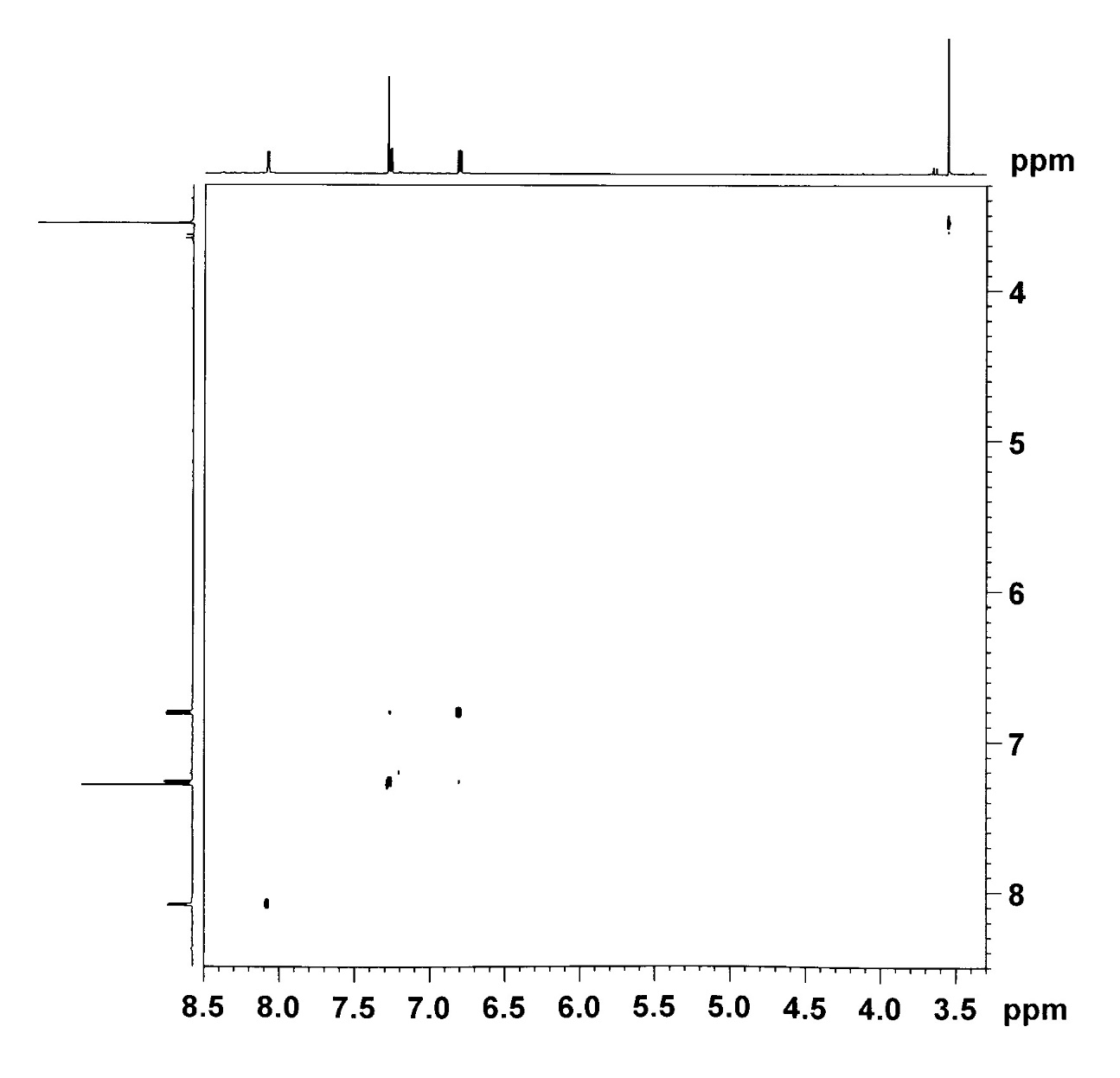


c. HSQC


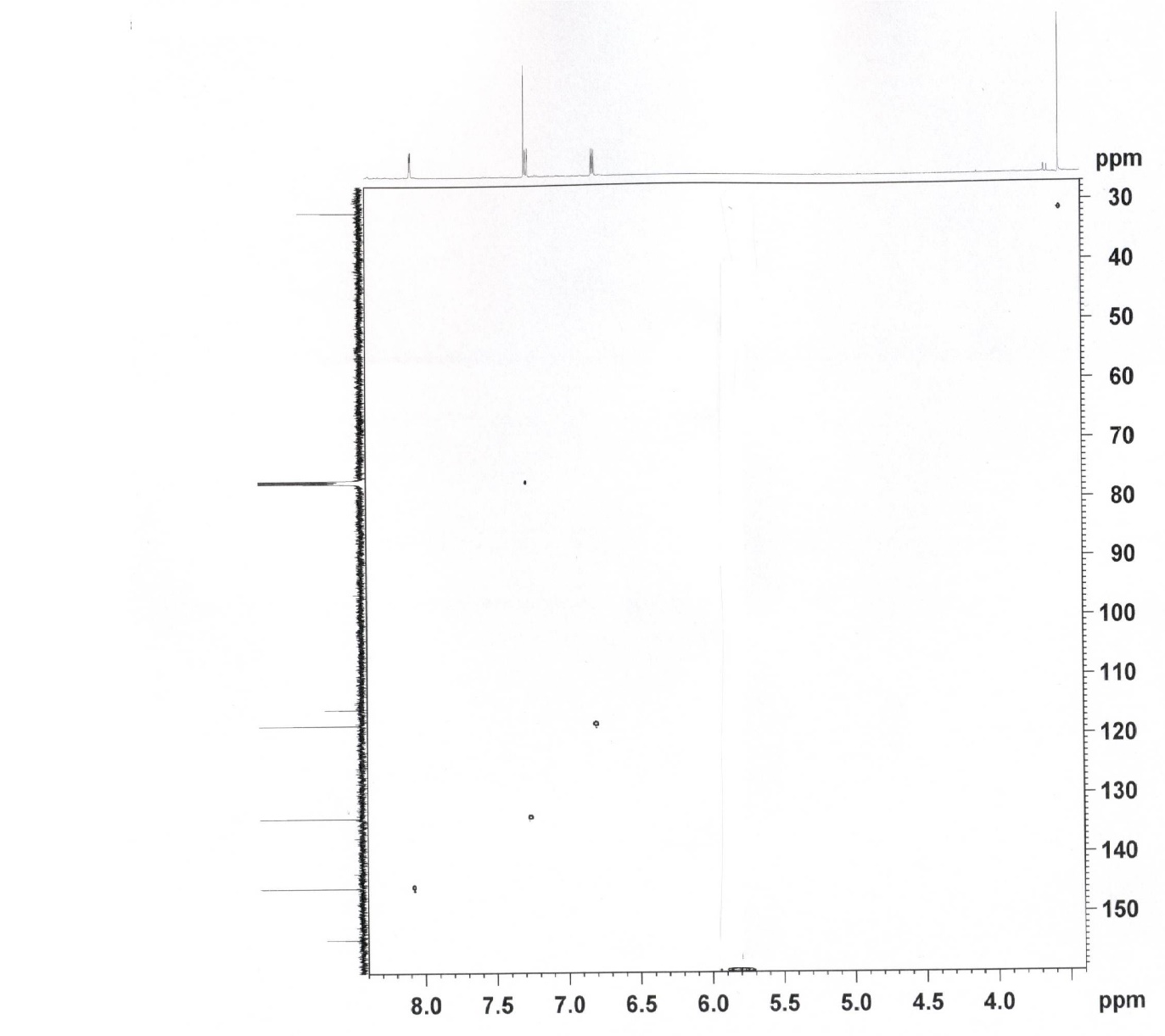


d. HMBC


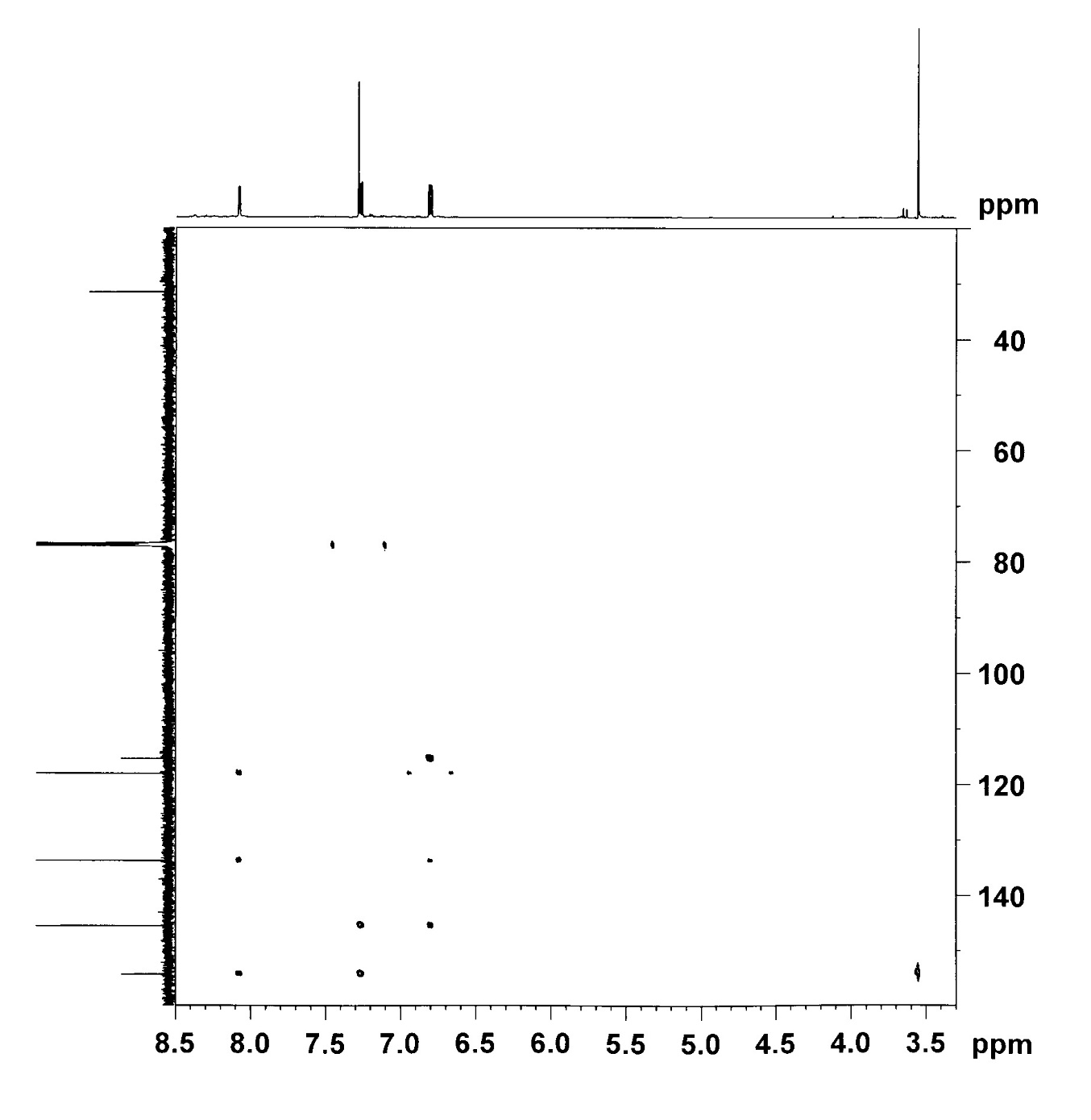


9. Table 1. The proton-proton and proton-carbon correlation in compound (**3**) (** **ppm)

| **^1^H NMR** | **NOESY** | **COSY** | **^13^C NMR** | **HSQC** | **HMBC** |
| --- | --- | --- | --- | --- | --- |
| 6.81 H_3,_ H_7,_  7.26 H_4_, H_6_, 8.08 H_2_, H_8_  3.45 CH_3_ | 6.81/7.26  7.26/6.81 | 6.81/7.26/8.08  7.26/8.08/6.81  8.08/7.26 /6.81 | 118.16 C_3_,C_7_, 133.83 C_4 ,_C_6_, 145.55 C_2,_C_8_  31.50 CH_3_  115.48 C_4a_, C_5a_  154.26 C_9a,_ C_10a_ | 6.81/118.16  7.26/133.83  8.08/145.55  3.45/31.50 | 6.81/115.48/133.83/145.55  7.26/145.55/154.26  8.08/118.16/133.83/154.26  3.45/154.26 |

10. EI MS of 10-methyl-1,9-diazaphenothiazine (**3**)


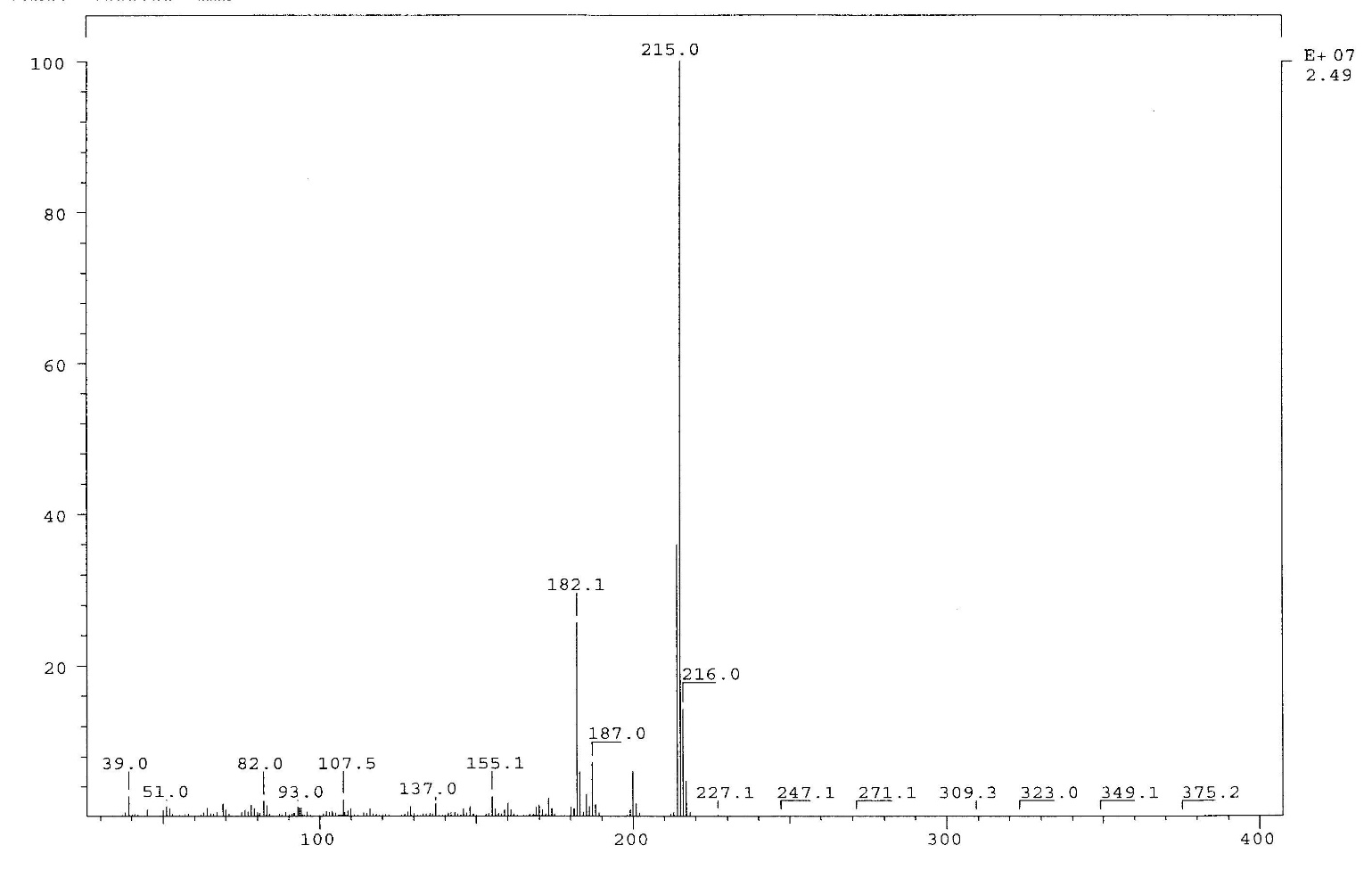


11. HR MS of 10-methyl-1,9-diazaphenothiazine (**3**)


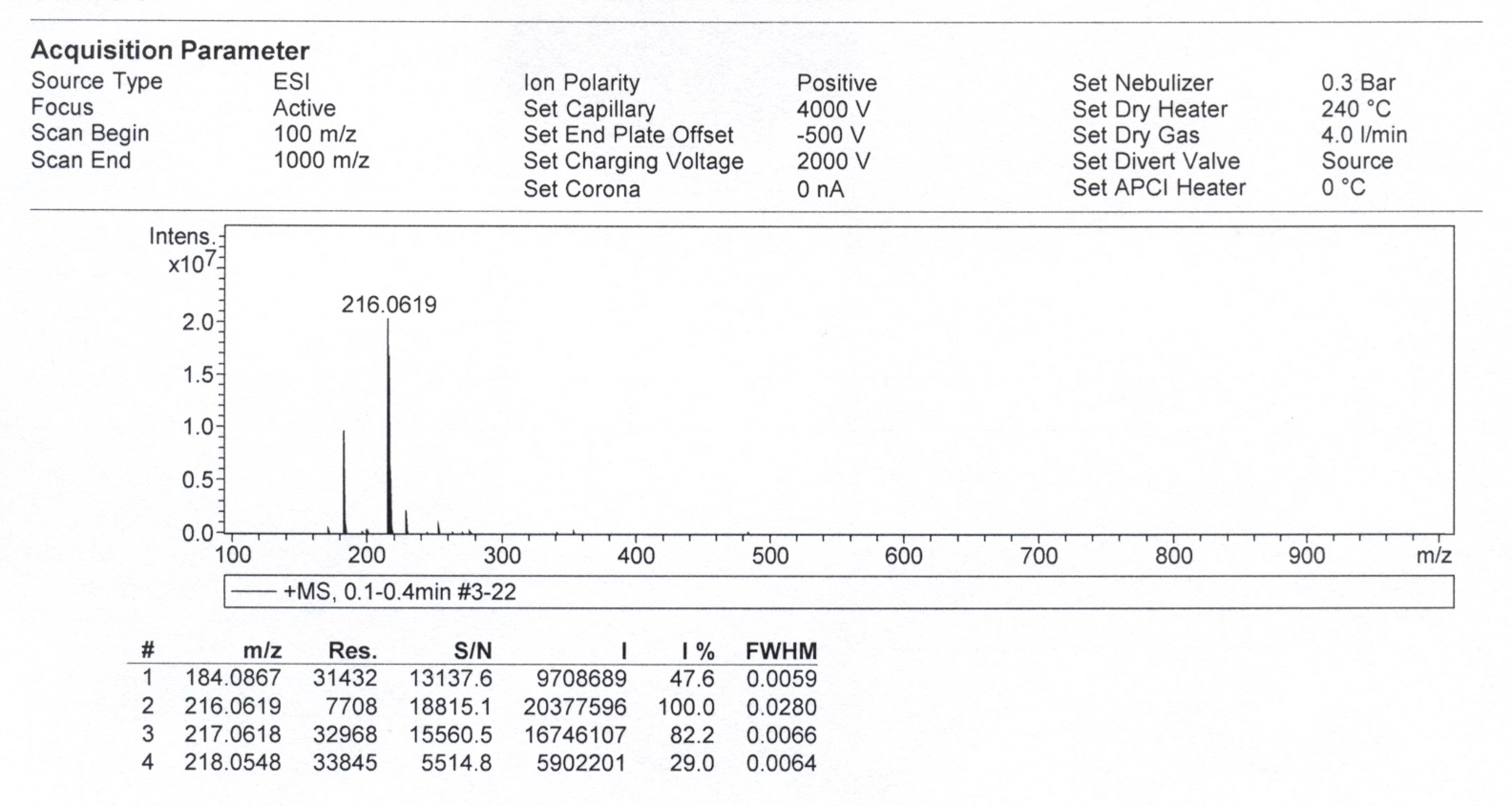


12. A layer-type structure of 10-methyl-1,9-diazaphenothiazine (**3**)


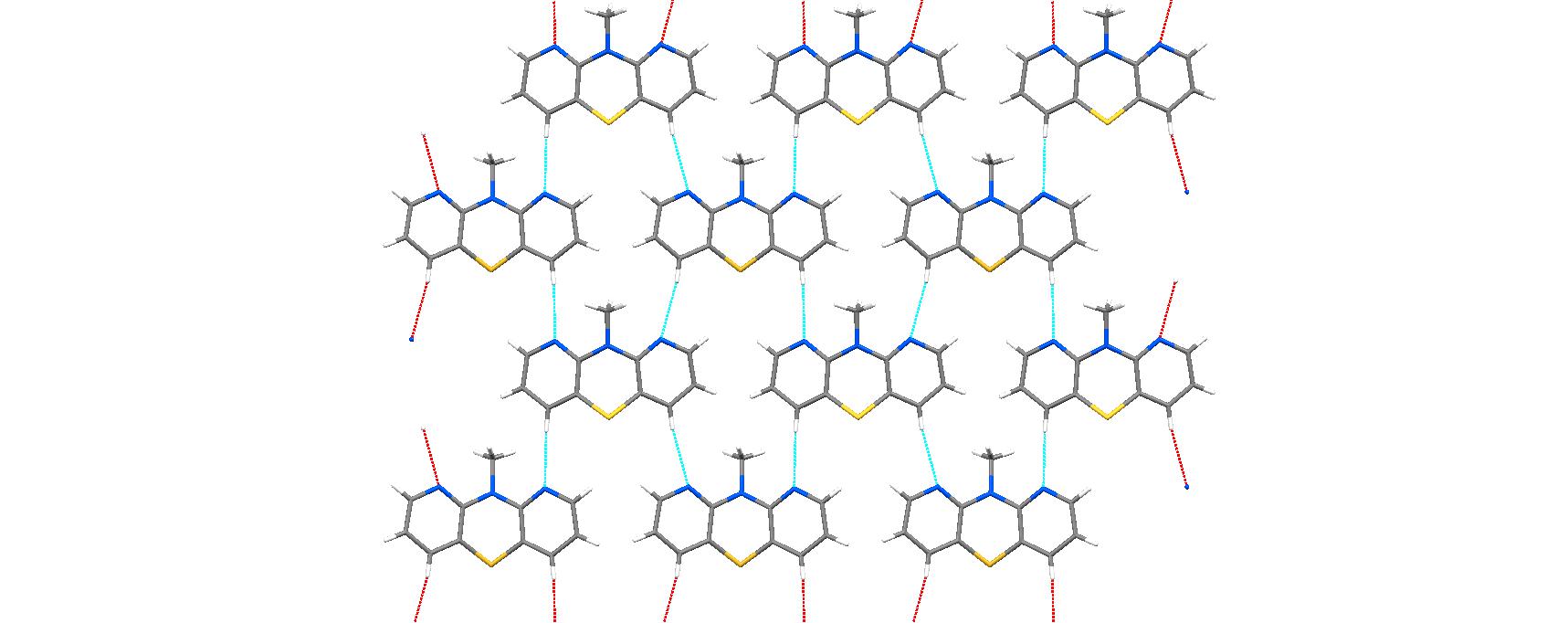


13. Proteome Profiler™ - compounds **5** and **8**


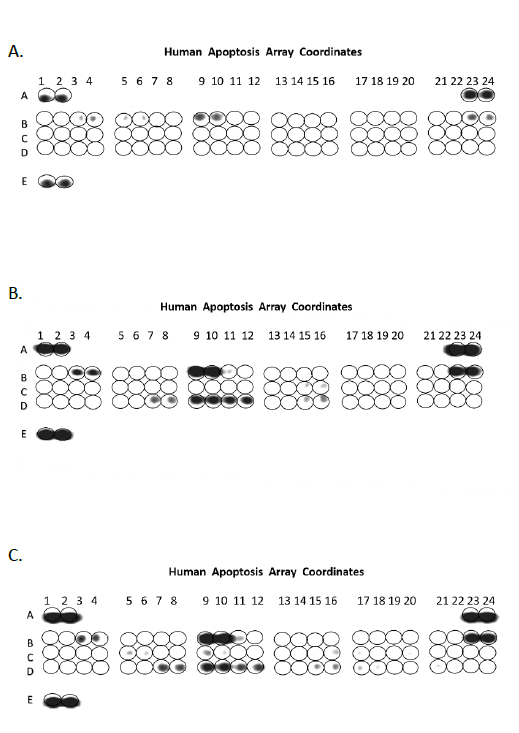


A – Control B - impact of compound **5** C- impact of compound **8**

**
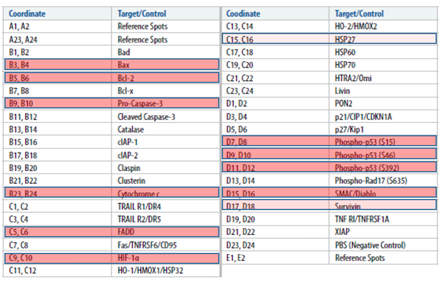
**

14. Cytometry (FCM) of compounds (**5)** and (**8**)


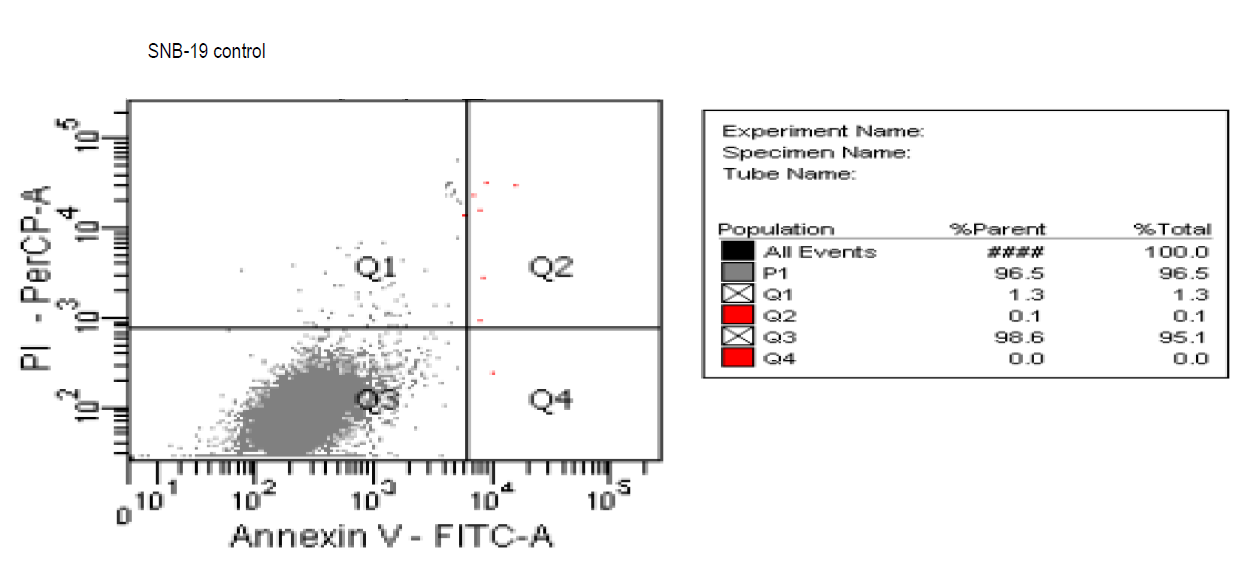


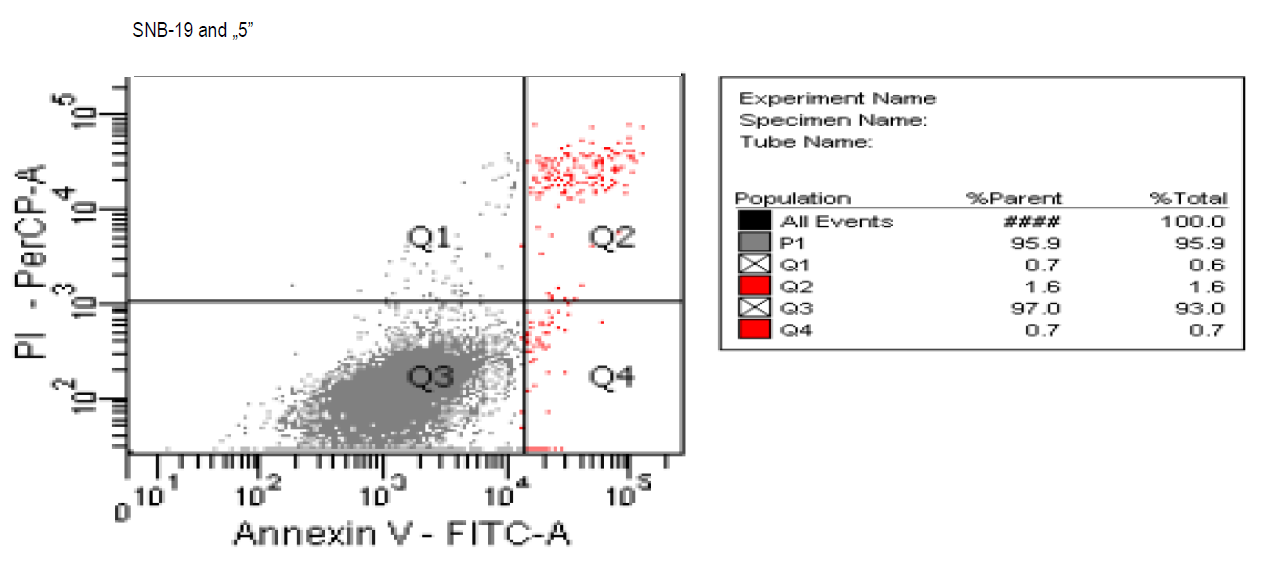


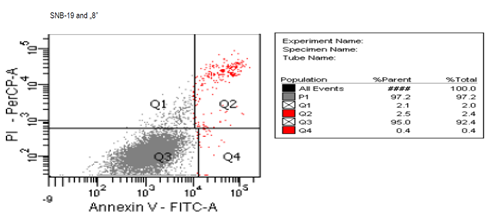

Supplement: Supplemental Material [file IENZ_A_1639695_SM9354.docx]
